# The Evolution Pathway of Ammonia-Oxidizing Archaea Shaped by Major Geological Events

**DOI:** 10.1093/molbev/msab129

**Published:** 2021-05-16

**Authors:** Yiyan Yang, Chuanlun Zhang, Timothy M Lenton, Xinmiao Yan, Maoyan Zhu, Mengdi Zhou, Jianchang Tao, Tommy J Phelps, Zhiwei Cao

**Affiliations:** 1 Department of Gastroenterology, Shanghai 10th People’s Hospital, School of Life Sciences and Technology, Tongji University, Shanghai, China; 2 Shenzhen Key Laboratory of Marine Archaea Geo-Omics, Southern University of Science and Technology, Shenzhen, China; 3 Southern Marine Science and Engineering Guangdong Laboratory (Guangzhou), Guangzhou, China; 4 Department of Biogeochemistry, Shanghai Sheshan National Geophysical Observatory, 201602 Shanghai, China; 5 Global Systems Institute, University of Exeter, Exeter, United Kingdom; 6 State Key Laboratory of Palaeobiology and Stratigraphy & Center for Excellence in Life and Paleoenvironment, Nanjing Institute of Geology and Palaeontology, Chinese Academy of Sciences, Nanjing, China; 7 College of Earth and Planetary Sciences, University of Chinese Academy of Sciences, Beijing, China

**Keywords:** ammonia-oxidizing archaea, nitrification, archaeal evolution, oxygenation, phylogenomic

## Abstract

Primordial nitrification processes have been studied extensively using geochemical approaches, but the biological origination of nitrification remains unclear. Ammonia-oxidizing archaea (AOA) are widely distributed nitrifiers and implement the rate-limiting step in nitrification. They are hypothesized to have been important players in the global nitrogen cycle in Earth’s early history. We performed systematic phylogenomic and marker gene analyses to elucidate the diversification timeline of AOA evolution. Our results suggested that the AOA ancestor experienced terrestrial geothermal environments at ∼1,165 Ma (1,928–880 Ma), and gradually evolved into mesophilic soil at ∼652 Ma (767–554 Ma) before diversifying into marine settings at ∼509 Ma (629–412 Ma) and later into shallow and deep oceans, respectively. Corroborated by geochemical evidence and modeling, the timing of key diversification nodes can be linked to the global magmatism and glaciation associated with the assembly and breakup of the supercontinent Rodinia, and the later oxygenation of the deep ocean. Results of this integrated study shed light on the geological forces that may have shaped the evolutionary pathways of the AOA, which played an important role in the ancient global nitrogen cycle.

## Introduction

Ammonia-oxidizing archaea (AOA) represent diverse ubiquitous nitrifiers accomplishing the first and rate limiting step of nitrification in today’s terrestrial and marine environments (Francis et al. 2005; Leininger et al. 2006; Stahl and de la Torre 2012; Qin et al. 2020), thus playing a major role in global nitrogen cycling. They were first reported in the early 1990s based on sequences of the *amoA* gene characteristic of the rate-limiting nitrifying enzyme ammonia-monooxygenase from the Marine Group I archaea (DeLong 1992; Fuhrman et al. 1992). AOA are now classified into the archaeal phylum Thaumarchaeota (Brochier-Armanet et al. 2008; Spang et al. 2010). Other extant nitrifiers belong to ammonia-oxidizing bacteria (AOB) that normally require higher ammonia levels (Prosser and Nicol 2012). In contrast, some AOA, especially strains from Group I.1a (such as *Nitrosopumilus maritimus* SCM1 [Konneke et al. 2005] and ca. *Nitrosoarchaeum koreensis* MY1 [Jung et al. 2011]), can grow at relatively much lower ammonia concentrations (Martens-Habbena et al. 2009; Hink et al. 2017; Kits et al. 2017).

Studying the evolution of AOA could shed light on the microbial nitrogen and carbon cycles in Earth’s early history (Godfrey and Falkowski 2009). Geochemical studies have estimated the earliest marine nitrification to be around 2.5–3.0 Ga (Garvin et al. 2009; Godfrey and Falkowski 2009; Zerkle and Mikhail 2017; Delarue 2018). Yet the lipid biomarker crenarchaeol for marine AOA was only found in the mid-Cretaceous (∼112 Ma) to early Triassic (245 Ma) ages (Kuypers et al. 2001; Saito et al. 2017). This apparent discrepancy between the geochemical evidence of nitrification and the occurrence of AOA based on fossil biomarker evidence begs for a new approach depicting the evolutionary changes of AOA.

In recent years, efforts using phylogenetics and molecular dating analysis have been employed to address AOA evolution. Gubry-Rangin et al. (2015) were the first to systematically study AOA evolution, focusing on *amoA* and 16S rRNA genes from the soil environment. They inferred that the ancestor of the mesophilic AOA clade (i.e., a group diverged from the thermophilic AOA group) likely emerged between 700 Ma and 1,400 Ma (Gubry-Rangin et al. 2015). Betts et al. (2018) integrated genomic and fossil evidence to derive a timescale of life’s early evolution in which the occurrence of marine AOA was around 570 Ma based on three representative strains. In addition, evidence of horizontal gene transfer suggested a divergence time window for the ancestor of AOA groups I.1a and I.1b between 950 and 750 Ma (Petitjean et al. 2012). Although these studies are all consistent in a general timeframe of AOA evolution, recent work estimates much older ages for major AOA clades (Ren et al. 2019).

To clarify the inconsistency between Ren et al. (2019) and other studies, we collected 90 archaeal and bacterial genomes covering a wide range of settings from hot springs, soils, freshwater and marine systems. The timeline of the AOA evolutionary pathway was investigated by phylogenomic analysis and was further validated by independent analysis of 33,378 *amoA* genes. Additionally, we linked evolutionary events identified by both the genome and *amoA* gene approaches with global geological events in Earth’s history, and we used geochemical modeling of the marine nitrogen cycle to try and understand the timing of AOA radiation into the deep oceans. The outcome of this integration of genetic analyses with geochemical modeling fills an important knowledge gap in microbial responses to major global events of glaciation and oxygenation in Earth’s early history.

## Results

### Thaumarchaeota Divergence Correlating with Major Global Events of Early Earth

**Fig. 1.**
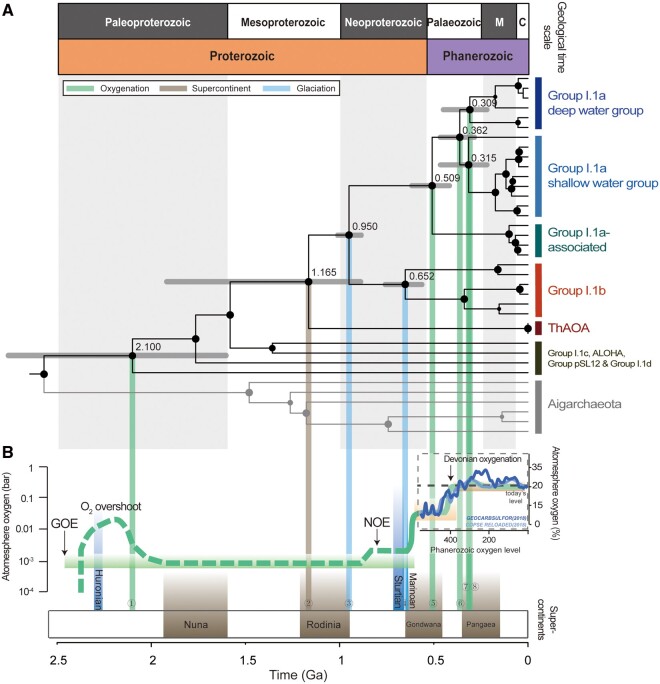
Timing correlation between key nodes of Thaumarchaeota and the major global events throughout the Earth’s history. (*A*) Thaumarchaeota phylogenomic time tree with 31 Thaumarchaeota genomes (with genomes of Aigarchaeota also shown here). The node sizes are proportional to the data coverages (defined as the proportion of sites where there is at least one taxon [sequence] in each descendent lineage that has available sequence data). The colored vertical bars on the right side indicate the groups of the Thaumarchaeota phylum. The divergence times of major nodes are numbered (#1–#8) and projected with color lines onto the time axis (green: oxygenation; brown: supercontinent; blue: glaciation). Time unit at the bottom is billions of years (Ga), which corresponds to the geological time periods in the top panel of figure 1*A* (M = Mesozoic; C = Cenozoic). (*B*) The Earth’s atmosphere oxygen curve and the major geological events of atmospheric oxygen, global glaciation and supercontinent events. The atmospheric oxygen’s partial pressure on the left is in bar, where 1 bar = 0.9869 atm. GOE = Great Oxidation Event, which started at ∼2.46–2.3 Ga (Gumsley et al. 2017; Warke et al. 2020) with its permanent atmospheric oxygenation at ∼2.22 Ga (Poulton et al. 2021), and was followed by the “Lomagundi event” including an oxygen overshoot at ∼2.22–2.06 Ga (Bekker and Holland 2012; Bachan and Kump 2015). NOE, Neoproterozoic Oxygenation Event (∼0.8–0.5 Ga). The atmospheric oxygen curve during the Phanerozoic is estimated by the GEOCARBSULFOR model (light blue) (Krause et al. 2018) and the COPSE reloaded model (dark blue) (Lenton et al. 2018). Glaciation events shown here are Huronian (2.29–2.25 Ga) (Tang and Chen 2013), Sturtian (717–659 Ma), and Marinoan (645–635 Ma). Figure 1*B* was modified from the oxygen curve in Catling and Zahnle (Catling and Zahnle 2020) and the major global events were based on Campbell and Allen (Campbell and Allen 2008).

The Thaumarchaeota phylogenomic subtree derived from an archaeal tree (see “The timeline and robustness testing of phylogenomic tree” section) was analyzed in detail. Timing of key nodes on the geological timescale is illustrated in figure 1, alongside atmospheric oxygen levels, global glaciations (Huronian, Sturtian, and Marinoan) and supercontinent events (Nuna, Rodinia, Gondwana, and Pangaea).

The Thaumarchaeota phylum contained non-AOA members: Group I.1c (Lin et al. 2015; Weber et al. 2015), Group I.1d (Beam et al. 2014), ALOHA (Mincer et al. 2007), and Group pSL12 (Reji and Francis 2020), which were commonly recognized as lacking the function of ammonia oxidation but having the potential ability of using oxygen. It also contained four AOA groups. The ThAOA group (thermophilic AOA group) contained organisms from hyperthermal settings; the Group I.1b contained representatives exclusively from soils and sediments; the Group I.1a-associated were from acidic soils; and the Group I.1a came from mostly marine water columns and sediments (see details in supplementary materials, Supplementary Material online). We chose their dominant environmental settings as the representative habitat for each group. Along the evolutionary pathway of the whole Thaumarchaeota phylum, the divergence times of key nodes (fig. 1*A*) were correlated to geological events (see details in “Major global events in Earth’s history” in supplementary materials, Supplementary Material online) in respective time windows (fig. 1*B*).

According to the dated genomic tree, the timing of the Thaumarchaeota root for both AOA and non-AOA groups (2.10 Ga, 94% CI: 2.77–1.59 Ga) (divergence node #1) may postdate the Great Oxidation Event (∼2.4 Ga) whose permanent atmospheric oxygenation was recently inferred at ∼2.2 Ga (Poulton et al. 2021) and correspond to the end of the subsequent “Lomagundi event” (2.10–2.05 Ga) (Bekker and Holland 2012; Bachan and Kump 2015; Hodgskiss et al. 2019). It almost certainly postdated the origin of oxygenic photosynthesis estimated at the earliest ∼3.0 Ga (Crowe et al. 2013; Planavsky et al. 2014).

The AOA lineages with the first indication of being capable of ammonia oxidation diverged from other non-AOA lineages around1.16 Ga (94% CI: 1.93–0.88 Ga), coinciding with the main collisional events resulting in the formation of the Rodinia supercontinent which took place at ∼1.1 Ga (ca. 1.2–1.0 Ga) (divergence node #2) (Martin et al. 2020). A large increase of global subduction-induced arc-volcanism and thermal activity resulted from the amalgamation of Rodinia (Liu et al. 2017), potentially creating a long-term suitable macro-environment for the origin of thermophilic traits in AOA ancestors, which have been kept in the modern ThAOA group.

About 900 Ma, Rodinia began to break apart. The unique middle Neoproterozoic paleogeography of a rifting, low-latitude Rodinia likely favored a globally cool climate due to the enhanced silicate weathering feedback and planetary albedo (Hoffman et al. 1998; Cox et al. 2016; Hoffman et al. 2017). The onset of climate cooling broadly coincided with the origin time of mesophilic traits (0.95 Ga, 94% CI: 0.88–1.02 Ga) (divergence node #3). This is supported by a recent study that dated glacial records back to the earliest Neoproterozoic (∼1.0 Ga) (Hartley et al. 2020).

There followed extreme global glaciations during the Neoproterozoic (Cox et al. 2016; McKenzie et al. 2016), which may have been the trigger for the origin of the first mesophilic terrestrial AOA clade Group I.1b (652 Ma, 94% CI: 554–767 Ma) (divergence node #4). Its central age was estimated in the interglacial period (659–645 Ma) shortly after the Sturtian glaciation (ca. 717–659 Ma), the longest-lasting and most severe glaciation in Earth’s history, and before the Marinoan glaciation (ca. 645–635 Ma) (Rooney et al. 2015). This may also have been a time of increased atmospheric oxygen levels due to the transition from dominantly bacterial to eukaryotic primary production (Brocks et al. 2017). The mesophilic environment created by glaciation near thermophilic settings (e.g., hot springs or geothermal vents) may have provided ecological niches for the evolutionary diversification of AOA (see below).

The common ancestor of Group I.1a (marine-dominated) and Group I.1a-associated (acid soil-dominated) lineages is estimated to have originated around 509 Ma (94% CI: 629–412 Ma) in the early Paleozoic (divergence node #5), with an uncertainty range spanning the Ediacaran-Cambrian-Ordovician-Silurian periods. This was an interval of rising and fluctuating oxygen levels, which culminated in the persistent oxygenation of the deep ocean (∼400 Ma). Because the Group I.1a-associated group was phylogenetically more similar to Group I.1a rather than to the mesophilic terrestrial Group I.1b, we infer that the habitat of this common ancestor was marine-related, from which Group I.1a-associated diverged and later adapted to acidic soil settings. After that, the estimated divergence of marine-dominated mesophilic Group I.1a (362 Ma, 94% CI: 478–274 Ma) (divergence node #6) occurred within the Paleozoic Era, with a best estimation around the Devonian-Carboniferous Era, a time of predicted rising atmospheric oxygen—the “Devonian oxygenation” (∼400 Ma) (Dahl et al. 2010; Krause et al. 2018; Lenton et al. 2018). This was followed by the radiation of each subgroup in shallow and deep waters at 315 Ma (94% CI: 478–207 Ma) and 309 Ma (94% CI: 460–207 Ma), respectively (divergence nodes #7 and #8). This broadly corresponds to the highest peak of atmospheric oxygen level (∼25–30% atm) estimated at ∼300 Ma by different oxygenation models (Hansen and Wallmann 2003; Bergman et al. 2004; Berner 2009; Arvidson et al. 2013; Mills et al. 2016; Krause et al. 2018; Lenton et al. 2018).

The phylogenetic analysis of the AOA genomes is further validated via the archaeal *amoA* gene database, considering that *amoA* marker gene targets the key enzyme for catalyzing the conversion of ammonia to nitrite in all AOA (see supplementary results, Supplementary Material online). According to the comparison between these two dated phylogenetic trees, the two key nodes in *amoA* gene tree of Group I.1b and Group I.1a & Group I.1a-associated branches still correlate with the glaciation and oxygenation events (supplementary fig. 1, Supplementary Material online), demonstrating the consistent findings between different data sets. A slight time difference was noted between the *amoA* gene tree and the AOA genomic tree. The reason could be due to their different data sizes as the *amoA* genes are much more diverse than the genomic data, thus being able to show greater resolution in the phylogenetic tree. On the other hand, the AOA genome data set can be easily integrated and compared with other non-AOA clades, thus some of their ancestors could be well calibrated and contribute to a more reliable tree. Nevertheless, the two key nodes of mesophilic terrestrial-related and marine-related branches still correlate with the glaciation and oxygenation events, demonstrating the consistent findings between different data sets.

In summary, our results demonstrated that throughout the Earth’s history, the key nodes in the adaptive process of AOA matched well in timing with the geological events, suggesting that the diversification within Thaumarchaeota, particularly the AOA lineages, may be shaped by global intercorrelated oxygenation, glaciation and supercontinent activities.

### Biogeochemical Modeling of AOA in Deep Oceans

From the above analysis, the marine-related common ancestor of Group I.1a and Group I.1a-associated originated at ∼509 Ma and the marine related Group I.1a at ∼362 Ma (94% CI: 478–274 Ma), which was likely followed by diversification of shallow-water and deep-water groups at around 315 Ma and 309 Ma, respectively. These age estimates are much later than the original rise of atmospheric oxygen at the Great Oxidation, but span the time when the deep ocean became persistently oxygenated. Oxygen and ammonium are the necessary substrates for nitrification, which control the distribution and activity of marine AOA in modern oceans (Qin et al. 2017). The better growth efficiency of AOA at low ammonium concentrations allows them to out-compete AOB in the deep ocean today. However, during the Proterozoic Eon and the Early Paleozoic Era, the deep ocean generally had large volumes of anoxic waters, whilst surface waters were oxygenated by equilibration with the atmosphere (Lenton and Daines 2017). Consequently, anoxic deep waters would have built up significant concentrations of ammonium (Lenton and Daines 2017). Where those waters came into contact with oxygen, it would have provided a niche for nitrification, which nitrogen isotope evidence indicates was occurring from ∼2.4 Ga onwards (Zerkle and Mikhail 2017). But what organisms performed that nitrification is uncertain. The physiology of extant nitrifiers suggests that AOB may originally have dominated a more ammonia-rich ocean. However, a recent study gives an estimated age of the oldest total group of AOB (Nitrosococcaceae) of 894 Ma (95% CI: 596–1,169 Ma) and the oldest crown group AOB (Nitrosomonadaceae) of 538 Ma (95% CI: 414–662 Ma) (Ward et al. 2021). Thus, whilst AOB may have predated AOA in the ocean, it is currently unclear what was nitrifying from ∼2.4 Ga onwards—as they may have left no descendants to the present. What is reasonable to hypothesize is that those earliest nitrifiers had lower affinity for ammonia than extant AOA, having adapted to an ammonia-rich ocean. We further hypothesize that the oxygenation of the deep ocean ∼0.6–0.4 Ga was sufficient to lower deep ocean ammonium concentration to levels where AOA could out-compete extant “low affinity nitrifiers (LAN)” for ammonium and radiate into the ocean.

**Fig. 2.**
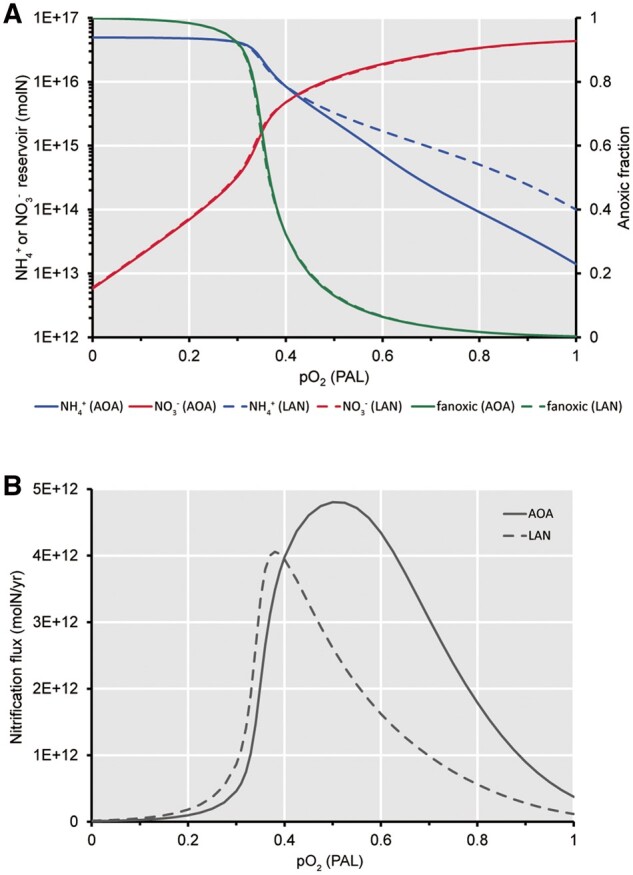
Steady state modeling results for ocean redox and nitrogen cycle as a function of atmospheric pO_2_ (present atmospheric level; PAL) assuming present levels of phosphate in the ocean. (*A*) Deep ocean ammonium (${\text{NH}}_{4}^{+}$, blue) and nitrate (${\text{NO}}_{3}^{–}$, red) reservoirs, and anoxic fraction of the ocean (green). (*B*) Corresponding deep ocean nitrification flux converting dissolved ammonium to nitrate, which shows at pO_2_ = 0.4 PAL the dominant nitrifying organisms (those achieving the higher flux and associated higher growth rate) switches between “low affinity nitrifiers” (LAN) and AOA in the case where both are present. Solid lines = AOA-only; dashed lines = LAN-only.

To illustrate the effect of deep ocean oxygenation on the relative success of AOA and “LAN,” a simple model was constructed of the deep ocean nitrogen reservoirs; ammonium (${\text{NH}}_{4}^{+}$) and nitrate (${\text{NO}}_{3}^{–}$); under varying ocean redox states (see Materials and Methods and supplementary fig. 2, Supplementary Material online). The model ocean redox state and associated nitrogen cycling were determined by prescribed atmospheric pO_2_ and ocean ${\text{PO}}_{4}^{3–}$ levels with the switch from an oxic to an anoxic deep ocean occurring at pO_2_/${\text{PO}}_{4}^{3–}$ ∼0.4 of their present concentrations based on more complex, spatially-resolved modeling (Lenton and Daines 2017). For simplicity, results are shown (fig. 2) by just varying atmospheric pO_2_ under present day ${\text{PO}}_{4}^{3–}$, noting that phosphorus levels over Earth history are uncertain, but proxies suggest they were at or above present in the Cryogenian-Ediacaran Periods (Planavsky et al. 2010; Reinhard et al. 2017) and models predict they were relatively stable over the Phanerozoic (Lenton et al. 2018).

Under low atmospheric pO_2_ that creates a largely anoxic deep ocean, organic nitrogen is mostly remineralized to ammonium, which hence is the dominant marine nitrogen species—as it was during the Archean and much of the Proterozoic Eon (Zerkle and Mikhail 2017). Under high atmospheric pO_2_ creating mostly oxygenated deep waters, most organic nitrogen is remineralized to nitrate, which hence dominates the modern oceans. Around pO_2_/${\text{PO}}_{4}^{3–}$ ∼0.4 an intermediate deep ocean redox state is predicted, with a mixture of deep ocean ammonium and nitrate pools (in the anoxic and oxygenated waters, respectively) both turning over rapidly, with widespread denitrification balanced by abundant nitrogen fixation—in good agreement with more complex models (Lenton and Daines 2017).

The simple model was run with the kinetics for AOA-only and separately with the kinetics for LAN based on contemporary AOB kinetics (see supplementary materials, Supplementary Material online). The only marked difference in the results was a higher-steady state ammonium concentration in a mostly oxygenated ocean with LAN (fig. 2*A*). When the model was run with AOA and LAN competing, there was a switch from LAN dominance at high ammonium to AOA dominance at low ammonium. This switch occurred (under pO_2_ = 0.4 PAL in fig. 2) at ∼8 × 10^15^ mol ${\text{NH}}_{4}^{+}$ (corresponding to [${\text{NH}}_{4}^{+}$] ∼6 μM), and was determined by the specified growth response curves (supplementary fig. 3, Supplementary Material online) (Prosser and Nicol 2012). In terms of Figure 2 the results follow the dashed lines to the left of pO_2_ = 0.4 PAL and the solid lines to the right. The nitrate and ammonium reservoirs become comparable in size at ∼6 × 10^15^ mol N under pO_2_/${\text{PO}}_{4}^{3–}$ ∼0.425, at which there is a predicted minimum in the total N content of the ocean ∼1.2 × 10^16^ mol N, that is, ∼28% of the present-day reservoir.

Modeling results showed that oxygenation of the deep ocean would have lowered deep ocean ammonium concentrations to levels (Prosser and Nicol 2012) of [${\text{NH}}_{4}^{+}$] ∼6 μM where AOA could have out-competed LAN for ammonium, allowing them to radiate into this massive niche. The greater growth efficiency of AOA at low ammonium levels (fig. 2*B*) would then have reinforced the transition from an ammonium- to a nitrate-dominated ocean, such that AOA today maintain deep ocean [${\text{NH}}_{4}^{+}$] ∼0.01 μM (Gruber 2008), whereas with just LAN it was predicted to be ∼7-fold higher (fig. 2*A*). Importantly the predicted transition to AOA-dominance corresponds to a mixed deep ocean redox state.

### The Timeline and Robustness Testing of Phylogenomic Tree

Eighty-three archaeal genomes and seven bacterial genomes (as outgroups) were used to calculate key occurrence times throughout the history of the Thaumarchaeota phylum (supplementary table 1, Supplementary Material online). Seventy conserved marker proteins derived from each genome were concatenated as one for phylogenetic tree construction (supplementary table 2, Supplementary Material online). Both RAxML (Stamatakis 2014) and IQ-Tree (Nguyen et al. 2015) were adopted for tree construction. The tree topology and branch lengths were highly similar (supplementary fig. 4, Supplementary Material online), suggesting the stability of the phylogenomic tree. The timeline of the archaeal phylogenomic tree was estimated by RelTime (Tamura et al. 2012; Tamura et al. 2018) with bacterial genomes as outgroups. Four calibration scenarios were performed based on root setting of a range of 4.38–3.46 Ga. Different scenarios gave similar node age estimations (<0.05 Ga in age difference). More details can be found in supplementary figure 5, Supplementary Material online.

**Fig. 3.**
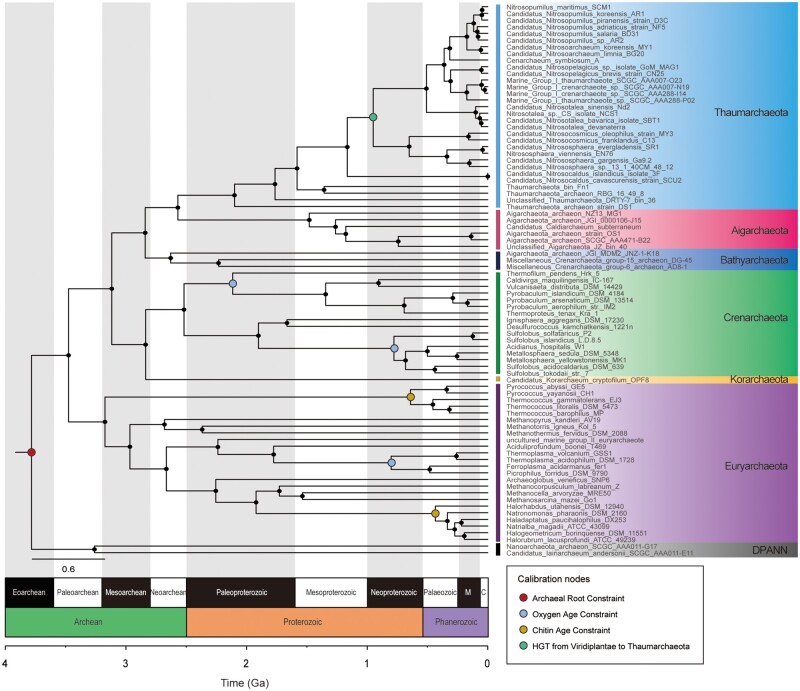
Phylogenomic tree of archaea based on an old archaeal root age constraint. The phylogenetic tree was constructed with 70 concatenated marker proteins using RAxML v8.2.10 and dated by RelTime v10.1.5. Seven bacterial genomes were used as outgroups, which were trimmed off and not shown in the tree. The colored bars indicate the major phyla in the Archaea domain with the exception of DPANN that was named as a superphylum. Calibration nodes are highlighted in colors to display different types of constraints for calibration with the rest nodes being small black dots. Here the Archaeal root constraint is set with a maximum of 4.38 Ga and a minimum of 3.46 Ga, the oxygen age constraint with a maximum of 2.32 Ga, the chitin age constraint with a minimum of 1.58 Ga, and the horizontal gene transfer constraint from Viridiplantae to Thaumarchaeota with a maximum of 1.49 Ga and a minimum of 0.75 Ga. Time unit is billions of years (Ga), corresponding to the geological time periods at the bottom (M = Mesozoic; C = Cenozoic).

Figure 3 displays the phylogenetic relationship of major phyla in the archaeal domain on the geological timescale (see more details in supplementary fig. 6, Supplementary Material online). Two strains belonging to the DPANN superphylum were the nearest to the archaeal root at around 3.8 Ga, and the phylogenomic tree identified the divergence of Euryarchaeota phylum followed by the TACK superphylum (namely Thaumarchaeota, Aigarchaeota, Korarchaeota and Crenarchaeota) with an additional Bathyarchaeota phylum being close to Aigarchaeota, displaying an overall similar tree topology to those presented by recent studies (Zhu et al. 2019; Abby et al. 2020; Baker et al. 2020). Furthermore, the Thaumarchaeota phylum diverged from Aigarchaeota and its crown age was ∼2.1 Ga, corresponding to the mid-Paleoproterozoic Era.

It is understood that different timing of a phylogenetic tree may be drawn based on different bioinformatics approaches, such as the selection of models or calibration nodes. Thus, we performed a robustness test by varying different influencing factors. Firstly, we investigated the potential impact of calibration effects on age calculations. Four types of calibration evidence were adopted, namely 1) archaeal root constraint, 2) oxygen age constraint, 3) chitin age constraint, and 4) HGT constraint from Viridiplantae to Thaumarchaeota. By simply reducing them one by one from all types to merely one type, the divergence times of the whole tree were recalculated. The results showed that perturbation of calibration gave consistent ages on major nodes, indicating that calibration effects unlikely to cause the great discrepancy (supplementary fig. 5, Supplementary Material online). To test the influence of oxygen-age constraint on the timeline, we also reset it to an older age of ∼2.5 Ga when a whiff of early oxygen may have occurred (Anbar et al. 2007). The divergence times of all nodes under this constraint are very similar to the original results (RSME = 0.0358 and two-side *t*-test *P*-value = 0.8789 > 0.05). A sensitive test by adding and subtracting 200 My to other types of constraints has also been performed, and the unanimous low RSMEs as well as high *P*-values (> 0.05) of *t*-test further indicate the robustness of our model (supplementary table 3, Supplementary Material online).

**Fig. 4.**
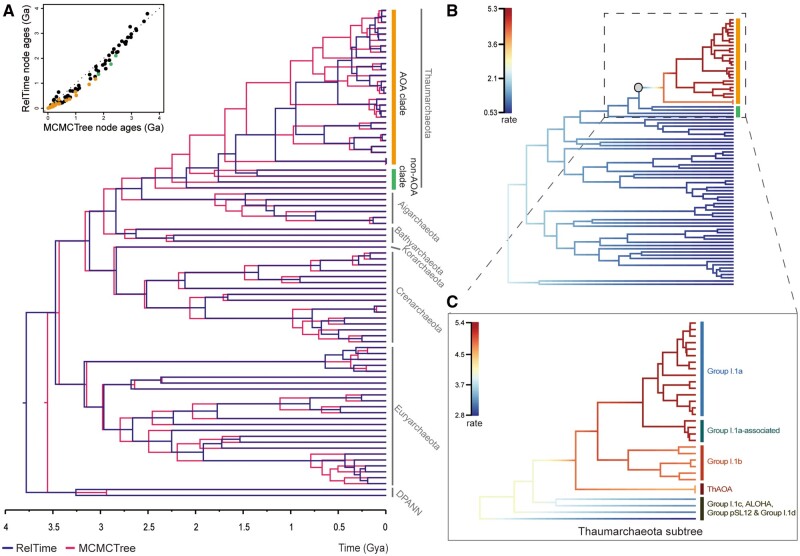
Comparison of the estimated divergence times using RelTime and MCMCTree approaches. (*A*) The RelTime (purple lines) and the MCMCTree (pink lines) trees overlapped along the geological timescale based on the archaeal genomic data, with bacterial outgroups being omitted from the figure. The vertical bar in orange highlights thaumarchaeotal species having the ammonia oxidation (AO) function, whereas the bar in green indicates those without the AO function and the remaining strains belong to other archaeal phyla. At the top left of figure 4*A* is the scatterplot of RelTime and MCMCTree estimated node ages; The orange dots correspond to the AOA clade, the green dots to the non-AOA clade within Thaumarchaeota, and the black dots to the clades from other Archaea in panel A. The overlapped trees demonstrate the consistently greater ages by the MCMCTree approach. (*B*) Phylorate plot of the genomic phylogenetic time tree showing evolutionary rates along each branch estimated by BAMM v2.5.0 (Rabosky and Matute 2013; Rabosky et al. 2013, 2014) (top). The gray circle node indicates a significant shift in evolutionary rate. The colored vertical bars on the right of the tree correspond to those in panel *A*. The most probable diversification rate shift configurations can be found in supplementary figure S14, Supplementary Material online. (*C*) The zoom-in and rescaled phylorate plot of the Thaumatcheaota. The colored bars indicate major groups in the Thaumarchaeotal phylum.

Then, the effects of different models were tested. By keeping the same data set and calibration points, RelTime was replaced by another classical model MCMCTree (Yang 2007). Interestingly, the molecular dating results showed an average of ∼30% older time on the key nodes of the Thaumarchaeota root, AOA root, ancestor of Group I.1a and deep-water group than that from RelTime (fig. 4*A*, supplementary fig. 7 and table 4, Supplementary Material online). This demonstrates that MCMCTree model intrinsically gives older time estimation than RelTime for the AOA data set. In recent years, the tendency of MCMCTree to produce older dates was noticed where major shifts occur in the evolutionary rates near the base of a clade, due to its attempts to fit the whole tree using the same branch rate model (like lognormal model) (Beaulieu et al. 2015; Mello et al. 2017). A close examination of the distribution of branch lengths demonstrated that the Thaumarchaeota phylum, particularly the AOA clade, are averagely 25% longer than that of non-AOA archaeal lineages (Kolmogorov–Smirnov test, *P*-value = 7.4*e*-07) (supplementary fig. 8, Supplementary Material online). This often suggests a significant rate heterogeneity in the phylogenomic tree and acceleration of the long-branch clade (Tamura et al. 2012). Further BAMM results confirmed our speculation of rate shift in the tree and slight acceleration for the AOA clade (fig. 4*B* and *C* and supplementary fig. 9, Supplementary Material online). This enhanced rate may compromise the accuracy of age estimation by MCMCTree. Thus, RelTime is recommended for molecular dating analysis on large data of sequences when rate heterogeneity is detected, such as in this case of the AOA clade.

## Discussion

The divergence times of AOA have been recently studied with great interest but haven’t been put into a unified and compatible dated framework with previous evidence (Petitjean et al. 2012; Gubry-Rangin et al. 2015; Betts et al. 2018; Ren et al. 2019). By performing phylogenomic and marker gene analyses integrated with geochemical modeling, we provided a reasonable and justified timeline of AOA evolution and its association with geological events. Our results demonstrated that selective Thaumarchaeota strains gained the ability of ammonia oxidation and the AOA clade diverged at around 1,165 Ma. The emerged AOA progressed through an adaptive pathway from terrestrial hot springs to mesophilic soil (∼652 Ma) and then to marine environments (∼509 Ma). Our ages support the previous publications derived from different evidence, such as mesophilic AOA hinted by Gubry-Rangin et al. (2015), marine Thaumarchaeota by Betts et al. (2018), and HGT event by Petitjean et al. (2012). Each of these key nodes along the AOA phylogenomic tree corresponds well with major global events of oxygenation, glaciation and supercontinent activities, demonstrating the connection between geological events and major AOA divergence times.

During the early history of AOA evolution, several major events might have played significant roles in shaping species from thermophilic settings to mesophilic ones. Among the supercontinents, Rodinia was characterized as having much more intense global subduction-induced arc-volcanism and thermal activity resulting from amalgamation (Liu et al. 2017). The amalgamation of Rodinia was a long-lived event but its core was formed around 1.1 Ga (1.2–1.0 Ga) (Martin et al. 2020). This coincides with the origination time of AOA at ∼1.16 Ga, suggesting it might have created conditions triggering the emergence of thermophilic traits in AOA and allowed them to gradually adapt and evolve into the new environments. About 900 Ma, Rodinia gradually transited into its breakup phase. The unique middle Neoproterozoic paleogeography of a rifting, low-latitude Rodinia likely favored a globally cool climate due to the enhanced silicate weathering feedback and planetary albedo (Hoffman et al. 1998; Cox et al. 2016; Hoffman et al. 2017). The start of climate cooling broadly coincided with the origin time of mesophilic-trait ancestors (0.95 Ga, 94% CI: 0.88–1.02 Ga), supported by a recent study that dated glacial records back to the earliest Neoproterozoic (∼1.0 Ga) (Hartley et al. 2020). With the ongoing climate cooling, the Earth entered into the Cryogenian Period 0.72 Ga.

The origin of Group I.1b (the first mesophilic terrestrial group) appears to be linked to the Cryogenian Period of “Snowball Earth” events and subsequent “Hothouse Earth” intervals. There is a wide acceptance that mesophilic AOA have thermophilic ancestors (Brochier-Armanet et al. 2012; López-García et al. 2015; Hua et al. 2018; Abby et al. 2020), and it has been proposed that the ability of AOA to adapt to mesophilic habitats was gained from bacteria via horizontal gene transfer (Deschamps et al. 2014; López-García et al. 2015; Nelson-Sathi et al. 2015). But what drove this transition is unknown. We proposed that temperature and chemical gradients were ubiquitously formed around the hot springs due to the global and long-lasting extremely cold surface environments during the Cryogenian Period, which may have created numerous high-to-low temperature gradient niches, as well as cultivation media, to allow the colonization of mesophilic lineages. This may be supported by a recent study, which reported that microbial diversification increased as the temperature decreased in a hot spring setting, implying that cold conditions might favor microbial evolution around thermal settings (Podar et al. 2020). Furthermore, each “Snowball Earth” episode was followed by temporary “hothouse Earth” conditions caused by high CO_2_ and low albedo after the ice melted (Hoffman et al. 1998; McKenzie et al. 2016). Perhaps the “hothouse” created conditions in which thermophilic AOA could leave thermogenic, for example, hot-spring settings and colonize suitable cooler terrestrial soils. This interglacial time period may also have experienced an increase in oxygen level due to the transition from dominantly bacterial to eukaryotic primary production (Brocks et al. 2017). In addition, the unique lipids of AOA might have contributed to the transition, because the AOA-specific biomarker crenarchaeol may adjust the membrane fluidity by forming a cyclohexane ring, and this dynamic variation in membrane structure may have enabled AOA to move from thermophilic to mesophilic habitats (Damsté et al. 2002; Zhang et al. 2006).

The timing of AOA evolving into marine settings is consistent with the well-established data that suggest atmospheric oxygenation preceded ocean oxygenation (Scheller et al. 2018). Regarding deep ocean redox conditions, the earliest evidence for widespread oxygenation of the deep ocean takes the form of trace metal data for a series of temporary “ocean oxygenation events,” starting after the Marinoan “Snowball Earth” ∼635 Ma (Sahoo et al. 2012), and continuing through the Ediacaran (635–541 Ma) and Cambrian (541–485 Ma) Periods (Sahoo et al. 2016). This builds on earlier iron-speciation redox proxy evidence for one of these ocean oxygenation events at ∼580 Ma (Canfield et al. 2007), in which the deep ocean redox state remained spatially-variable with one deep ocean basin oxygenated and other parts of the deep ocean remaining anoxic (Canfield et al. 2008; Li et al. 2018). According to our model, such partial deep ocean oxygenation might have triggered a spread of AOA into the deep ocean, broadly consistent with our age range for AOA ancestor of Group I.1a and Group I.1a-associated first entering marine habitats 629–412 (∼509) Ma. As these ocean oxygenation events were temporary, LAN could have recolonized when more widespread anoxic conditions returned with associated accumulation of ammonium.

The permanent establishment of deep ocean AOA lineages that have persisted to the present presumably had to await the persistent oxygenation of the deep ocean. Current data and models estimate that this occurred later in the Paleozoic Era associated with the rise of land plants during the Ordovician-Silurian-Devonian Periods ∼420–360 Ma (Lenton et al. 2016; Krause et al. 2018; Lenton et al. 2018). This is broadly consistent with our estimated divergence of Group I.1a ∼362 (478–274) Ma. Models then tend to predict an ongoing rise and peak in atmospheric (and oceanic) oxygenation around 300 Ma at the boundary of the Carboniferous-Permian Periods (Krause et al. 2018; Lenton et al. 2018). This is broadly consistent with our estimated radiation times for shallow-water and deep-water groups at around 315 (478–207) Ma and 309 (460–207) Ma, respectively. Following the Permian-Triassic boundary and during the subsequent Mesozoic Era there was partial deoxygenation of the deep ocean in occasional “oceanic anoxic events” (OAEs). However, the estimated expansion of anoxia (typically of order 10% of the deep ocean) (Clarkson et al. 2018) was presumably insufficient to allow ammonium to build up to levels that could have caused expansion of AOB to eliminate AOA lineages from the deep ocean.

Our model predictions of N cycling under different deep ocean redox states compare well to results from more complex, spatially resolved modeling (Lenton and Daines 2017) (see supplementary materials, Supplementary Material online). The conditions under which AOA is predicted to take over from LAN, with a mix of anoxic and oxic deep ocean waters containing ${\text{NH}}_{4}^{+}$ and ${\text{NO}}_{3}^{–}$ respectively, requires a special balance of atmospheric oxygen and ocean phosphate levels (Lenton and Daines 2017). Hence, it would be expected to have been a transient feature of Earth history, between largely anoxic ocean conditions that characterized the Proterozoic and largely oxic ocean conditions that characterize the later Phanerozoic. In contrast, an alternative simple box model of the nitrogen cycle (Fennel et al. 2005; Johnson and Goldblatt 2015) predicts the Proterozoic deep ocean had substantial reservoirs of both ${\text{NO}}_{3}^{-}$ and ${\text{NH}}_{4}^{+}$ even under a low prescribed Proterozoic O_2_ of 1% of present atmospheric levels. However, that model fails to represent most of the oxygen consumption processes in deeper waters, erroneously predicting a deep ocean O_2_ concentration close to equilibrium with the atmosphere (fig. 5 of Johnson and Goldblatt 2015). Instead, under low Proterozoic O_2_ the deep ocean should be anoxic and dominated by ${\text{NH}}_{4}^{+}$ (Lenton and Daines 2017), consistent with geochemical evidence.

Finally, we would like to emphasize that the critical ages for AOA’s major transitional points in our work and others (Petitjean et al. 2012; Gubry-Rangin et al. 2015; Betts et al. 2018) are much younger than those presented in Ren et al. (2019). In particular, the major difference between our study and the Ren et al. (2019) appears to be the choice of model, with similar discrepancies between models also observed in other studies (Tamura et al. 2012; Mello et al. 2017). Here, we chose RelTime because it doesn’t require the same statistical distribution of rates and can better tolerate the rate heterogeneity along the tree (Mello et al. 2017). And based on the observation of longer branch lengths of the AOA clade than the non-AOA archaeal lineages, we further identified the rate heterogeneity in the phylogenomic tree. On the other hand, Ren et al. (2019) used the classical Bayesian model to fit the same branch rate (e.g., lognormal) to the whole tree, which might result in outstandingly old times.

In summary, our timeline of AOA diversification, in accordance with most previous studies, agreed well with geological events in early Earth’s history. We are aware that, though AOA were reported to have descended from a thermophilic Thaumarchaeotal ancestor, this hypothesis requires further testing because of the placement of new mesophilic Thaumarchaeota genomes within the Nitrosocaldales/ThAOA clade by a recent study (Sheridan et al. 2020). Nevertheless, our findings should serve the purpose of encouraging future studies that integrate more comprehensive genetic approaches with geochemical evidence or modeling in addressing ancient microbial processes co-evolving with the Earth system.

## Materials and Methods

### Construction of the Phylogenomic Tree

Ninety representative genomes, including eighty-three archaeal genomes and seven bacterial genomes as outgroups (supplementary table 1, Supplementary Material online), were collected and downloaded from NCBI genome database (https://www.ncbi.nlm.nih.gov/genome, last accessed on 12 July 2019) and IMG/M database (https://img.jgi.doe.gov/, last accessed on 13 July 2019) to construct the genomic phylogenetic tree. Seventy conservative and homologous proteins (supplementary table 2 and data 1, Supplementary Material online) in each genome were aligned by COBALT v2.1.0 (Papadopoulos and Agarwala 2007) and concatenated as input sequences of 12,082 bp (supplementary data 2, Supplementary Material online) for constructing maximum likelihood phylogenomic tree by RAxML v8.2.10 (Stamatakis 2014) (supplementary data 3, Supplementary Material online). Further details are provided in the supplementary methods, Supplementary Material online. 

### Estimation of the Phylogenomic Tree Timeline

Two approaches, RelTime v10.1.5 (Tamura et al. 2012; Mello et al. 2017; Tamura et al. 2018; Tao et al. 2020) and MCMCTree v4.8 (Yang 2007), were performed to estimate the origination times of key nodes within the Thaumarchaeota phylum (Sanderson 2003). Seven nodes in the tree were calibrated using four calibration types. With either an old or a young range of root node, different types of calibration evidence could be combined resulting in a total of eight calibration scenarios to determine the possible ranges of nodes (supplementary table 5, Supplementary Material online). Further details are provided in the supplementary methods, Supplementary Material online.

### Construction of the *amoA* Gene Evolutionary Tree

An archaeal *amoA* gene data set was collected from a previous study, which contains 1,190 nonredundant archaeal *amoA* OTUs representing 33,256 sequences covering a wide range of environmental distribution of available *amoA* genes (Alves et al. 2018). To better represent *amoA* genes in species level and reduce the computational consumption, sequences were further unified at a cutoff of 90% and aligned resulting in an alignment of 241 OTUs (supplementary data 4, Supplementary Material online). With the aligned sequences, the *amoA* gene tree was constructed by RAxML v8.2.10 (Stamatakis 2014) under GTR+G+I model (supplementary data 5, Supplementary Material online). The time line of the *amoA* gene tree was estimated by RelTime v10.1.5. The root node was calibrated with the same calibration ranges (minimum = 750 Ma; maximum = 1,487 Ma) for the AOA node in the phylogenomic tree. Further details are provided in the supplementary methods, Supplementary Material online.

### Nitrogen Cycle Model

Our nitrogen cycle model simulates the deep ocean nitrate (${\text{NO}}_{3}^{–}$) and ammonium (${\text{NH}}_{4}^{+}$) reservoirs, and is forced by prescribed levels of deep ocean phosphate (${\text{PO}}_{4}^{3–}$) and atmospheric oxygen (pO_2_). The details of the model are provided in supplementary methods, Supplementary Material online.

### Diversification Rate and Shift Analysis

Evolutionary rate and major rate shift detection of the phylogenomic tree were simulated by BAMM v2.5.0. The BAMM model assumes that phylogenetic trees are shaped by a countable set of distinct and potentially dynamic evolutionary processes of speciation and extinction, where the estimated diversification rate (also often referred to as net diversification rate) is the difference between speciation rate (*λ*) and extinction rate (*μ*) explicitly inferred from a compound Poisson process (namely equals to *λ* – *μ*) (Rabosky and Matute 2013; Rabosky et al. 2013). The outputs were analyzed by a downstream R package BAMMtools v2.1.6 (Rabosky et al. 2014).

## Supplementary Material

Supplementary data are available at *Molecular Biology and Evolution* online.

## Supplementary Material

msab129_Supplementary_Data
