# Supplementary material for: The Evolution Pathway of Ammonia-Oxidizing Archaea Shaped by Major Geological Events
Source: Mol Biol Evol. 2021 May 16;38(9):3637–48. doi: 10.1093/molbev/msab129 (PMC8382903; doi:10.1093/molbev/msab129)
Supplement: msab129_Supplementary_Data [file msab129_supplementary_data.zip › Yang et al._Supplementary_Information.pdf]

Supplementary Information

The evolution pathway of ammonia-oxidizing archaea shaped by major geological events

Yiyan Yang†, Chuanlun Zhang†*, Timothy M. Lenton, Xinmiao Yan, Maoyan Zhu, Mengdi Zhou, Jianchang Tao, Tommy J. Phelps, Zhiwei Cao*

†Equal contribution

*Corresponding authors:

Chuanlun Zhang (zhangcl@sustc.edu.cn)

Zhiwei Cao (zwcao@tongji.edu.cn)

Materials and Methods

Genomic data

Fasta files of 90 genomes (Archaea 83, Bacteria 7 as outgroups) were collected and downloaded from NCBI Genome database (https://www.ncbi.nlm.nih.gov/genome) and IMG/M database (https://img.jgi.doe.gov/) before 11th August, 2019, along with their corresponding annotation .gff files (Supplementary Table 1). The reference genomes from NCBI Genome sequences were preferentially collected until they could cover the genera and groups within Archaeal domain. For those genera/groups not included in the references, we further searched the NCBI Assembly Database and even in the IMG/M database to obtain additional genomes. In the end, to balance the data coverage and computational consumption, 83 Archaeal sequences were finally included in this study, with 59 reference genomes, 9 assembly genomes and 17 IMG/M genomes (including 10 single-cell amplified genomes (SAGs) and 7 metagenome-assembled genomes (MAGs)). It should be noted that some strains were kept for the reason of their MRCAs being used as calibration points in future analysis. In addition, 5 bacterial genomes representing 5 main phyla within the Bacteria domain (namely, Actinobacteria, Proteobacteria, Aquificae, Chlamydiae/Verrucomicrobia, Thermotogae, Spirochaetes, Cyanobacteria) were selected as outgroups. As for the Thaumarchaeota, a total of 31 genomes covering all genera within this phylum were collected (with 17 reference genomes, 6 assembly genomes, and 8 IMG/M genomes). For those genomes coming from IMG/M database, they met the criteria of an estimated completeness ≥ 50% (except for Marine Group I thaumarchaeote sp. SCGC AAA288-P02 (IMG_2524023104) with 49.0 %) and an estimated contamination ≤ 10% (except for Miscellaneous Crenarchaeota group-15 archaeon DG-45 (IMG_2654588083) with 13.0 %) by using CheckM v1.1.1 (Parks et al. 2015) (Supplementary Table 7).

The genomes collected can be mainly divided into 4 groups based on both genomic data and *amoA* genetic data: 1) Candidatus Nitrosocaldales (a.k.a. ThAOA group, or NC group) present in thermophilic hot spring environment, 2) Nitrososphaerales (a.k.a Group I.1b, or NS group) present mostly in river sediments and soil, 3) Candidatus Nitrosotaleales (a.k.a. Group I.1a-associated or NT group) present in acid soils, and 4) Nitrosopumilales (a.k.a Group I.1a or NP group) present mostly in marine (the majority), soil and sponge-associated environments. In the Nitrosopumilales group, except for five strains, namely strains BD31 (estuary sediments), BG20 (estuary sediments), AR2 (soil), MY1 (marine sediments) and C. Symbiosum (sponge), members in this group all come from marine water settings.

Seventy marker proteins were the homologous proteins existing both in Archaea and Bacteria, with 32 being widely used as conservative ribosomal proteins and the other 38 being newly found marker proteins (Petitjean et al. 2015) (Supplementary Table 2 and Supplementary Data 1). All the encoded genes were extracted from genome sequences according to the annotation files. With each of 70 marker gene amino acid sequences queried, all the proteins (from 90 genomes) were searched against the PSI-BLAST (with evalue = 1e-05 and max_target_seqs = 1e7) to obtain homologous genes in each genome. PSI-BLAST was used here because it is an advanced Blastp program with higher sensitivity and often useful to find similar proteins or new members in a distantly related protein family (Altschul et al. 1997). However, for some marker proteins with short length (i.e. 50S ribosomal protein L29 is only 68-bp long), the e-value for these marker genes might be too strict resulting to limited mapping results, so one more step was added to further search for all the subjects by their names, making sure they were also the homologous proteins to the corresponding query.

Subsequently, each set of homologous proteins was aligned by COBALT v2.1.0 webserver (Papadopoulos and Agarwala 2007) (https://www.ncbi.nlm.nih.gov/tools/cobalt/) with rps blast constraint E-value = 0.003 and alphabet = 'Regular' and others remaining default. The resulting alignments were trimmed by BMGE v1.12 (Criscuolo and Gribaldo 2010) with parameter t = AA and others remaining default. All the marker genes in one genome were concatenated as the final input sequence of 12,082 bp for the construction of phylogenomic tree (Supplementary Data 2).

Phylogenomic tree and the time line of Thaumarchaeota

Forty-one partitions for seventy marker genes and corresponding best models for each partition were determined by PartitionFinder v2.1.1 (Lanfear et al. 2017) (Supplementary Table 6). A maximum likelihood (ML) tree was produced for concatenated data by RAxML v8.2.10 and

bootstrapped for 100 times (Supplementary Data 3) (Stamatakis 2014) with the parameters "-T 6 -f a -x 7 -p 7 -# autoMRE -k -m PROTGAMMALGX" to set thread number, seed, substitution model and bootstrap replicates. The bootstrap search was automatically stopped after 100 replicates according to the MRE-based bootstopping criterion in RAxML program (Supplementary Figure 6). In order to confirm the tree topology and branch lengths, IQ-Tree v1.6.12 (Nguyen et al. 2015) was also applied with the parameters "-st AA -m LG+I+G4+F -bb 1000 -alrt 1000" to set amino acid substitution model and 1,000 ultrafast bootstrap analysis as well as single branch tests. The comparison of these two results with Phylo.io (Robinson et al. 2016) and the congruency index calculated by icong (de Vienne et al. 2007) was shown in Supplementary Figure 4. Next, RelTime in MEGA X v10.1.5 (Tamura et al. 2012; Mello et al. 2016; Tamura et al. 2018; Tao et al. 2020) and MCMCTree in PAML v4.8 (Yang 2007) were performed to estimate the key node divergence times in the phylogenomic tree (Supplementary Figure 10A,B), with MCMCTree being run two times to ensure the convergence (Supplementary Figure 11).

It is well known that building an evolutionary tree with time often needs fossil evidence to calibrate nodes. But the information for microbiology remains limited. Blank has advocated that the rough time range of key nodes could be inferred by known global events and gene gain or loss (Blank 2009b; Blank 2011). In order to make the Thaumarchaeota divergence times more accurate and reliable, we calibrated seven more nodes on the tree based on four calibration types according to evidence from literature (Supplementary Table 5):

1. Using all the calibration nodes (called "all_calibrations");
2. Using calibration nodes except for the HGT constraint of Group I.1b and Group I.1a & Group I.1a-associated (called "no_Thaumarchaeota");
3. Using calibration nodes except for the divergence time of Group I.1b and Group I.1a and other three ancestral nodes having the genes underlying chitin degradation by ancestral state reconstruction analysis (called "no_chitin_Thaumarchaeota");
4. Only using calibration for archaeal root node (called "archaeal_root_only").

Combined with either an old or a young root calibration range to calibrate key nodes, a total of eight calibration scenarios (old or young root × 4 calibration combinations) were designed, which are 1) old_root + all_calibrations, 2) old_root + no_Thaumarchaeota, 3) old_root + no_chitin_Thaumarcheaota, 4) old_root + archaeal_root_only, 5) young_root + all_calibrations, 6) young_root + no_Thaumarchaeota, 7) young_root + no_chitin_Thaumarcheaota, and 8) young_root + archaeal_root_only.

Calibration points

Seven nodes on the phylogenomic tree were calibrated.

a) **The root node of Archaea domain.** An old range of 4.38-3.46 Ga and a young range of 3.8-2.7 Ga were assigned to this node. The old root scheme (4.38-3.46 Ga) was employed in a recent study of timing the evolution of methanogens based on horizontal gene transfer (Ueno et al. 2009; Valley et al. 2014; Wolfe and Fournier 2018). And the young root scheme (3.8-2.7 Ga) proposed in (Blank 2009a; Blank 2011) was also adopted.

b) **Three clades with ancestral nodes under oxygen age constraint.** The clades of *Thermoproteales*, *Sulfolobales* and *Thermoplasma*, whose genomes contain modern taxa that use oxygen as a terminal acceptor. It is inferred by ancestral state reconstruction analysis that the ancestors of these clades should have already metabolized oxygen. Also, due to their growing pH (0.7-2.0), they were thought to live in habitats that prevented the growth of cyanobacteria, which cannot grow below pH 5.5, in a co-existing local environment (Blank 2009b). Therefore, in order to use oxygen, these clades must have originated after the rise in atmospheric oxygen, and thus can be constrained to have originated after the GOE (i.e. 2.32 Ga) (Bekker et al. 2004).

c) **Two clades with ancestral nodes under chitin age constraint.** The clades of *Thermococcales* and *Halobacteriales* in genomes used in this study have modern taxa with chitinase genes in their genomes. And ancestral state reconstruction supports the presence of chitinases in the ancestors of the clades (Blank 2011). Therefore, these clades must have originated after chitin-producing organisms first appeared. Chitin metabolism is presumably constrained by the origin of chitin in opisthokonts which was thought to be 0.904-1.579 Ga (Parfrey et al. 2011; Eme et al. 2014). In other words, a maximum age constraint of 1.579 Ga can be assigned to these clades.

d) **The ancestral node of Group I.1b and Group I.1a clades calibrated by HGT from Viridiplantae to Thaumarchaeota.** A study on the evolutionary history of fused gene encoded DnaJ-Fer protein revealed that this gene was horizontally transferred from to the ancestor of Thaumarchaeota Group I.1a and Group I.1b, more precisely before the divergence of these two lineages but likely after their separation from the ThAOA group (Petitjean et al. 2012). According to fossil record and molecular dating estimates, the divergence of Viridiplantae from the two other Archaeplastida lineages occurred ~1.487 Ga (Parfrey et al. 2011) whereas the diversification of Viridiplantae started ~0.75 Ga (Douzery et al. 2004). The HGT from Viridiplantae to Thaumarchaeota occurred most likely during this time window, so the divergence of the Group I.1a and Group I.1b in Thaumarchaeota and their diversification could be set in the range of 1.487-0.75 Ga.

Major global events in Earth's history

The GOE (Great Oxidation Event) was reported to be ~2.46-2.3 Ga (Gumsley et al. 2017; Warke et al. 2020) and the oxygen overshoot ~2.22-2.06 Ga (Bekker and Holland 2012; Bachan and Kump 2015). The NOE (Neoproterozoic Oxygenation Event) was reported to be ~0.8-0.5 Ga (Och and Shields-Zhou 2012). The atmospheric oxygen curves during the Phanerozoic had two major peaks at ~400 Ma and ~300 Ma, respectively, which were estimated by the GEOCARBSULFOR model (Krause et al. 2018) and the COPSE reloaded model (Lenton et al. 2018). The three glaciation events are 1) Huronian (2.29-2.25 Ga) (Tang and Chen 2013), 2) Sturtian(717-659 Ma) (Rooney et al. 2015; Hoffman et al. 2017), and 3) Marinoan (645-635 Ma) (Rooney et al. 2015; Hoffman et al. 2017). The four supercontinent events included Nuna, Rodinia, Gondwana and Pangaea (Campbell and Allen 2008).

Phylogenetic time tree with *amoA* marker genes

An archaeal 1,190 *amoA* gene dataset was provided in Alves' work and derived from a collective and complete dataset of 33,378 *amoA* sequences and covered a wide range of environmental categories of available *amoA* genes (Alves et al. 2018). These 1,190 sequences were dereplicated at a 90% cut-off using the scripts of "pick_otu.py" and "pick_rep_set.py" provided by QIIME v1.9.1(Caporaso et al. 2010), resulting in a representative dataset of 241 OTUs of 594 bp in length as the input of constructing maximum likelihood phylogeny (Supplementary Data 4). These sequences were also assigned taxonomy by the criterion provided by Alves' work using the script "assign_taxonomy.py" with "-m mother" implemented in QIIME. The OTUs were next aligned by the script "align_seqs.py" in QIIME using an external reference database of 1180 aligned sequences from Pester's work (Pester et al. 2012) (with "-p 0.01 -e 0"). The alignment was further manually checked by MEGA X v10.1.5 (Kumar et al. 2018).

For *amoA* gene sequences, the best substitution model, GTR+G+I, with estimated base frequencies and with no partition was suggested by PartitionFinder v2.1.1 for building a maximum-likelihood (ML) tree. The *amoA* gene tree was constructed by RAxML v8.2.10 (Supplementary Data 5). According to the tree, *amoA* genes were clustered into four major groups which are NC group (corresponding to ThAOA in 16S-23S rRNA gene phylogenies of AOA), NS group (corresponding to Group I.1b), NT group (corresponding to Group I.1a-associated) and NP group (corresponding to Group I.1a) (Alves et al. 2018). The root node in the *amoA* gene tree (namely the MRCA of NS group and NP group) was calibrated by the same range (i.e. minimum = 750 Ma; maximum = 1487 Ma) as that for the MRCA of Group I.1b and Group I.1a in the phylogenomic tree. According to results of the phylogenomic tree, the ancestral node ages of

both NS group and NP/NT group were calibrated to be no more than 950 Ma. It is worth mentioning that the root of the *amoA* gene tree was suggested to be put between the NS group and NP/NT group by the MAD (minimal ancestor deviation) method (Tria et al. 2017) when excluding the NC group (Supplementary Figure 12A). However, it is observed that the NC strains was clustered as a group in our constructed ML tree (Supplementary Data 5). And when including NC group, the MAD analysis showed that the entire NC group had the minimal ancestor deviation in the tree, indicating this group is the closest to the root (Supplementary Figure 12B). Therefore, the NC group was selected as outgroups here. In accordance with the protocol in that of phylogenomic tree, RelTime v10.1.5 was also adopted to estimate the time line of *amoA* gene tree.

Nitrogen cycle model

Our model (Supplementary Figure 2) simulates the deep ocean nitrate (NO_3^-) and ammonium (NH_4^+) reservoirs, and does not consider their surface ocean reservoirs (accepting that the surface ocean contains a significant fraction of the total ocean ammonium reservoir today, but that it turns over extremely rapidly). The model is forced by prescribed levels of deep ocean phosphate (PO_4) and atmospheric oxygen (pO_2), noting that phosphorus has a longer residence time (~20 kyr) than nitrogen (~5 kyr) in today's ocean, and atmospheric oxygen has a much longer residence time (~7 Myr) than either. Both dynamic reservoirs are in molN and all fluxes are in molN yr^{-1}. Reservoir sizes and fluxes are based on the COPSE model where possible (Lenton et al. 2018).

Key model processes are export production sinking from the surface ocean (E_{Total}) fuelled by the upwelling of nitrate (E_{NO3}) and ammonium (E_{NH4}) and by in-situ nitrogen fixation (E_{NFix}). The deep ocean is divided into an anoxic fraction (f_{anox}) and an oxic fraction ($1–f_{anox}$). Sinking export production is re-mineralised in the oxic fraction of the deep ocean to nitrate (R_{ox}) and in the anoxic fraction to ammonium (R_{an}). Ammonium is converted to nitrate by nitrification (F_{Nit}) and nitrate is lost to the atmosphere by denitrification (F_{DeN}). The model constants can be found in Supplementary Table 8. We exclude the burial of nitrogen species as this is a small flux occurring predominantly on continental shelves and the model focuses on the deep ocean (note that including a small burial fraction has minimal quantitative effect on the results).

Differential equations

$$\frac{dNH_4}{dt} = R_{an} - E_{NH4} - F_{Nit}$$

$$\frac{dNO_3}{dt} = R_{ox} + F_{Nit} - E_{NO3} - F_{DeN}$$

Process equations

Export production fuelled by nitrogen fixation:

$$E_{NFix} = k_{NFix} \cdot \left(\frac{16 \cdot PO_4 - (NO_3 + NH_4)}{k_{def}}\right)$$

Where k_{NFix} = 8.6x10^{12} molN yr^{-1} is today's ocean nitrogen fixation flux, PO_4 (molP) is the prescribed ocean phosphate content, which today is 3.1x10^{15} molP, and k_{def} = 6.086x10^{15} molN is the present day deficit of N below P required by primary producers with Redfield ratio (N:P=16) in the global ocean.

Export production fuelled by upwelling nitrate:

$$E_{NO3} = k_{mix} \cdot k_{uptake} \cdot NO_3$$

Where k_{mix} = 0.006 yr^{-1} is a global ocean upwelling rate (Tyrrell 1999), and k_{uptake} = 0.5 is a global efficiency of conversion of upwelled nutrient into export production (Lenton and Daines 2018).

Export production fuelled by upwelling ammonium:

$$E_{NH4} = k_{mix} \cdot k_{uptake} \cdot NH_4$$

These three components of export production are combined:

$$E_{total} = E_{NFix} + E_{NO3} + E_{NH4}$$

Anoxic fraction of the deep ocean is determined by the balance of export production (oxygen demand) and atmospheric oxygen (oxygen supply):

$$f_{anox} = \frac{1}{1 + e^{-k_{anox}\left((E_{total}/E_{max}) - O_2\right)}}$$

Where O_2 (in multiples of the present atmospheric level; PAL) is prescribed, k_{anox} = 12 determines the sharpness of transition between oxic and anoxic deep ocean (Lenton and Daines 2018), and E_{max} = 2.69684x10^{14} molN yr^{-1} is the maximum export production that could be supported today if all nitrate and ammonium were converted to export. This gives today's anoxic fraction f_0 = 0.00299.

Aerobic remineralisation:

$$R_{ox} = (1 - f_{anox}) \cdot E_{total}$$

Anaerobic remineralisation:

$$R_{an} = f_{anox} \cdot E_{total}$$

Denitrification:

$$F_{DeN} = k_{DeN} \cdot \left(1 + \frac{f_{anox}}{f_0}\right) \cdot \left(\frac{NO_3}{k_{NO3}}\right)$$

Where $k_{DeN} = 4.3\text{x}10^{12}$ molN yr^{-1} and $k_{NO3} = 4.35\text{x}10^{16}$ molN is the present day nitrate reservoir. For nitrification we have the same functional form but different kinetics for AOA and low affinity nitrifiers (LAN):

$$F_{Nit}^{i} = k_{Nit}^{i} \cdot k_{NH4} \cdot (1 - f_{anox}) \cdot \frac{NH_4}{K_m^i + NH_4}$$

Where $k_{NH4} = 1.4\text{x}10^{13}$ molN is the present day deep ocean NH_4 reservoir (based on a concentration of ~0.01 µM and ocean mass $1.4\text{x}10^{21}$ kg), nitrification only occurs in the presence of oxygen (represented by the oxic fraction of the ocean), k_{Nit} is the rate constant for nitrification, and K_m is the half-saturation constant for nitrification.

Nitrification by AOA has an observed half-saturation constant of ~0.15 µM which corresponds to $K_m^{AOA} = 2.1\text{x}10^{14}$ $molNH_4$, and we set $k_{Nit}^{AOA} = 0.429$ yr^{-1} in order to achieve a steady state deep ocean $NH_4 = 1.4\text{x}10^{13}$ $molNH_4$ today. It should be noted that this is much slower kinetics than achievable by AOA in the laboratory but represents an average value throughout the deep ocean.

Nitrification by low affinity nitrifiers (LAN) is based on extant AOB, with a much higher observed half-saturation constant ~8.5 µM corresponding to $K_m^{LAN} = 1.19\text{x}10^{16}$ $molNH_4$, but following Prosser and Nicol (2012) we reason that AOB must have a higher maximum growth rate than AOA (otherwise AOB would be out-competed everywhere, which they are not) (Prosser and Nicol 2012) and we set it roughly 2.5 times higher at $k_{Nit}^{LAN} = 1$ yr^{-1}.

Initially we ran the model with the kinetics for AOA then we ran it again with the kinetics for low affinity nitrifiers (based on contemporary AOB). The key difference in the results was a higher-steady state ammonium concentration in an oxic ocean with low affinity nitrifiers. Otherwise the results are very similar. For the results shown in the main paper (Fig. 2) we ran the model with the following switch:

$$F_{Nit} = max\left(F_{Nit}^{AOA}, F_{Nit}^{LAN}\right)$$

With the chosen parameters, the crossover point from LAN dominance at high NH_4 to AOA dominance at low NH_4 (determined by the growth response curves) occurs at $8.55\text{x}10^{15}$ $molNH_4$ equivalent to ~6.1 M (broadly consistent with Prosser and Nicol 2012).

We solve the model using Euler's method with a 1 year time step, integrating for up to 10,000 years to achieve steady state.

Steady state results

In Figure 2 of the main paper we show results for varying pO_2 at the present day $PO_4 = 3.1x10^{15}$ molP. The switch from an oxic to an anoxic deep ocean is determined by the ratio pO_2/PO_4 and occurs at pO_2/PO_4 ~0.4 of their present concentrations (Lenton and Daines 2017). This is also the level at which the switch between LAN and AOA dominance is predicted to occur. The nitrate and ammonium reservoirs become comparable in size (at ~$6x10^{15}$ molN) under slightly more oxygenated conditions; pO_2/PO_4 ~0.425. This corresponds to a predicted minimum in the total N content of the ocean ~$1.2x10^{16}$ molN, i.e. ~28% of the present day reservoir.

Results

The *amoA* gene analysis supporting the phylogenomic results

The phylogenetic analysis of the AOA genomes is further validated via the archaeal *amoA* gene database, because *amoA* marker gene targets the key enzyme for catalyzing the conversion of ammonia to nitrite in all AOA. Among 33,378 archaeal *amoA* sequences, 241 OTUs at a 90% cut-off were used to construct the phylogenetic tree, followed by calibration and molecular clock dating. The ThAOA clade was treated as the outgroup by the MAD (minimal ancestor deviation) method (Tria et al. 2017) (Supplementary Figures 12A, 12B).

The evolutionary pathway and timing of key nodes are displayed in Supplementary Figures 1. With the *amoA* genetic data, it is shown that Group I.1b, Group I.1a-associated and Group I.1a diverged in succession along the *amoA* gene tree, which agrees well with the evolutionary pathway observed in the phylogenomic tree. Further analysis showed decreases in GC content and the ratios of two charged amino acids along the ThAOA and Group I.1b lineages and the marine lineages, suggesting an adaptational trend of *amoA* gene from thermophilic to mesophilic conditions (Supplementary Figure 13).

The Group I.1b had an estimated age of 736 Ma based on the *amoA* genes, which is 84 million years older than the age estimated based on the genomic data; the Group I.1a & Group I.1a-associated had an estimated age of 481 Ma, which is 28 million years younger than the age estimated based on the genomic data. Despite the differences, the deviation falls within the original 94% CIs (8.87% for Group I.1b and 2.96% for Group I.1a & Group I.1a clades).

A slight time difference was noted between the *amoA* gene tree and the AOA genomic tree. The reason could be due to their different data sizes as the *amoA* genes are much more diverse than genome data, thus being able to show greater resolution in the phylogenetic tree. On the other hand, AOA genome dataset can be easily integrated and compared with other non-AOA clades, thus some of their ancestors could be well calibrated and contribute to a more reliable tree. Nevertheless, the two key nodes of mesophilic soil-related and marine-related branches still correlate with the glaciation and oxygenation events, demonstrating the consistent findings between different datasets.

Discussion

Comparison to GENIE results

The simple model predictions can be compared to a simulation of nitrogen cycling under a mixed ocean redox state with the 3D GENIE ocean model (Figure 6 of Lenton and Daines 2017).

The GENIE simulation set $pO_2 = 0.1$ PAL as an indicative Proterozoic level (Daines et al. 2017) and lowered PO_4 to achieve a mixed ocean redox state with a largely anoxic Pacific and a marginally oxic Atlantic basin. Whereas without a nitrogen cycle, $PO_4 = 0.25$ POL (present ocean level) produces such a mixed ocean redox state in GENIE, with a nitrogen cycle, PO_4 had to be doubled to 0.5 POL to achieve the same export production and deep ocean redox state. This is at least partly because nitrogen fixation is limited by temperature and light as well as nutrients in GENIE (Monteiro et al. 2012), hence available nitrogen tends to stabilise well below Redfield ratio (N:P=16) to phosphate. GENIE then gives predicted ocean inventories of 1.24×10^{15} $molNO_3$ and 2.96×10^{15} $molNH_4$ (total 4.2×10^{15} molN), supported by nitrogen fixation of 2.77×10^{13} molN yr^{-1}.

For a comparative simulation with our simple model we set $pO_2 = 0.1$ PAL and found that PO4 = 0.28 POL achieved f_{anox} ~0.5. In this state, predicted ocean inventories are 8.37×10^{14} $molNO_3$ and 3.36×10^{15} $molNH_4$ (total 4.2×10^{15} molN), supported by nitrogen fixation of 1.37×10^{13} molN yr^{-1} (which contributes ~50% to export production).

Given that no effort was made to tune the setup of the simple model to match the GENIE setup (beyond the choice of a function for f_{anox} that loosely matches where GENIE switches between oxic/anoxic ocean) this is an encouraging comparison. The main difference is that the balanced nitrogen fixation and denitrification fluxes are smaller in the simple model than in GENIE, presumably due to an underestimate of the increase in the global denitrification flux, which occurs at the interface of anoxic and oxic waters.

As with all simple models the results should be viewed as 'semi-quantitative' and aimed at getting the correct order of magnitude. Nevertheless, our simple model captures the key features of the more complex spatial model.

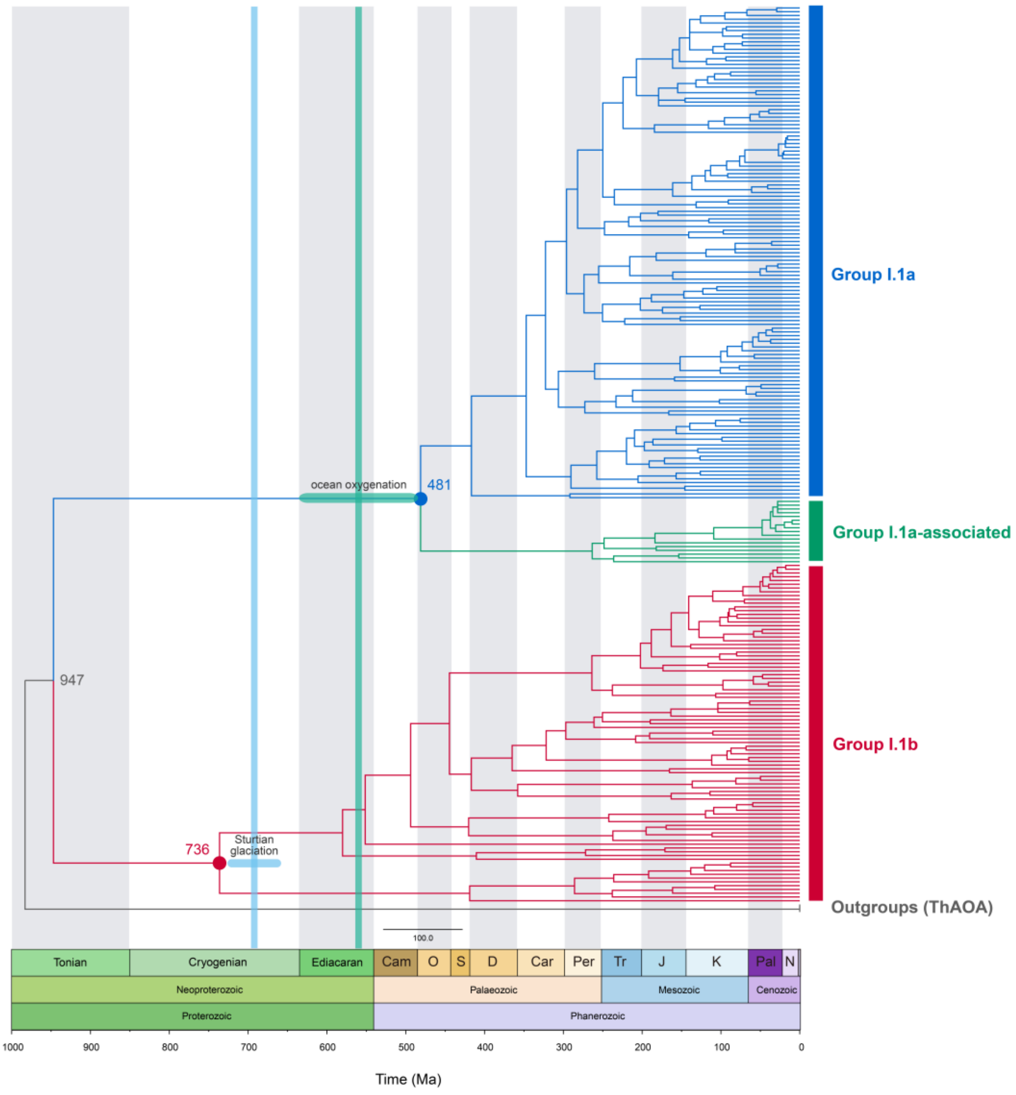

Fig. S1. The *amoA* gene tree timeline associated with global events in Earth's history. Four major clades of AOA were labeled, and ThAOA strains were used as outgroups. Time unit is millions of years (Ma), with the lower box indicating the corresponding geological timescale. The light blue vertical line indicates the Sturtian glaciation and the aqua blue one the ocean oxygenation event. The gray bars and the white blank alternate to show different periods of time.

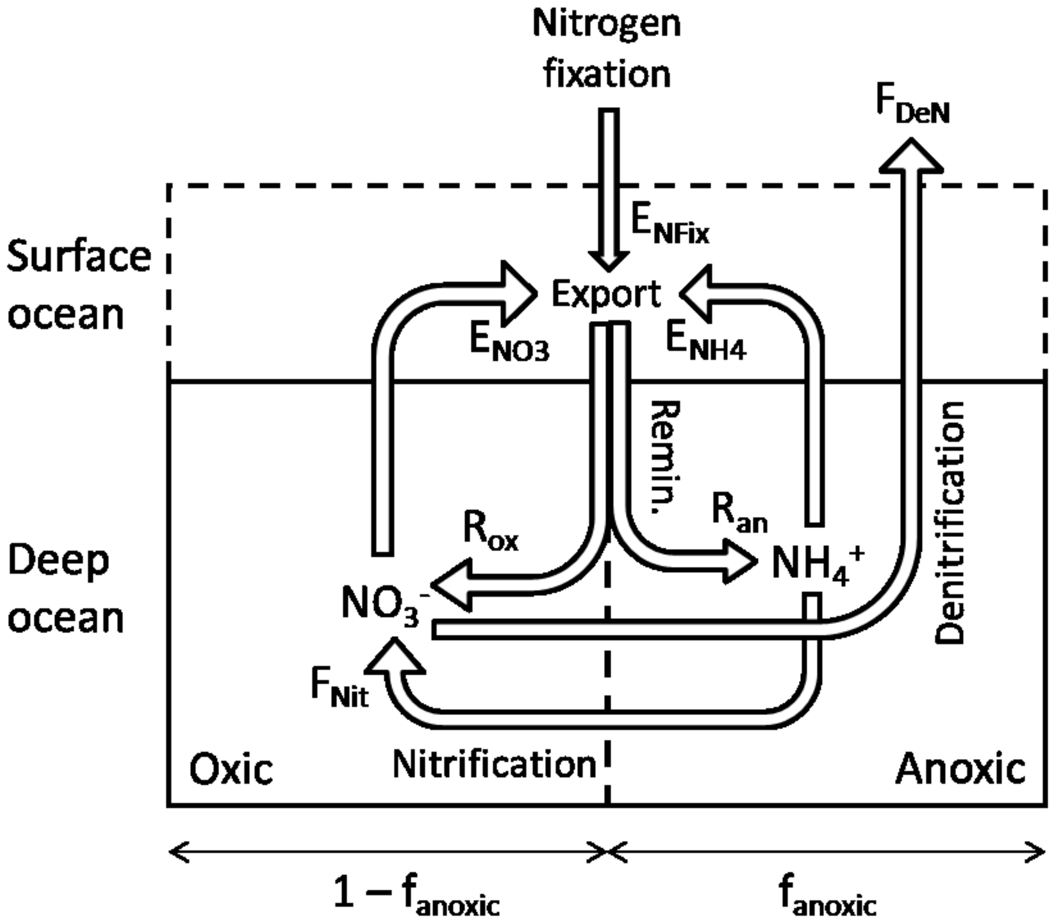

Fig. S2. Model schematic. The model considers deep ocean nitrate (NO_3^-) and ammonium (NH_4^+) reservoirs. The deep ocean is divided into an anoxic fraction (f_{anox}) and an oxic fraction ($1 - f_{anox}$). Total export production sinking from the surface ocean (E_{Total}) is fuelled by the upwelling of nitrate (E_{NO3}) and ammonium (E_{NH4}) and by in-situ nitrogen fixation (E_{NFix}). It is re-mineralised in the oxic fraction of the deep ocean to nitrate (R_{ox}) and in the anoxic fraction to ammonium (R_{an}). Ammonium is converted to nitrate by nitrification (F_{Nit}). Nitrate is lost to the atmosphere by denitrification (F_{DeN}). The model constants can be found in Table S8.

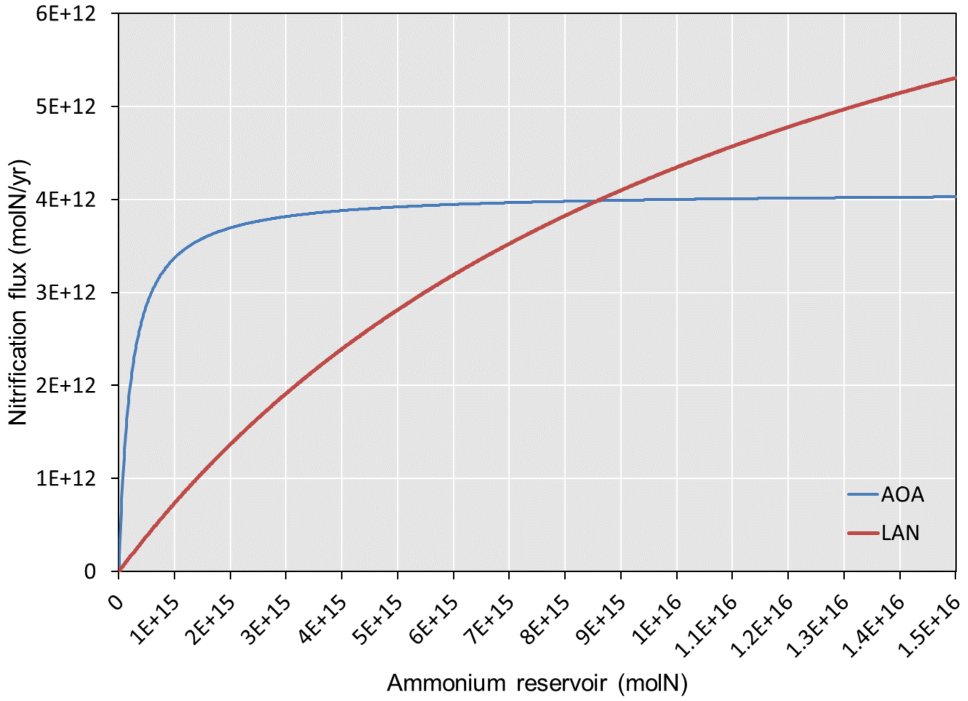

Fig. S3. Resource competition between ammonia-oxidising archaea (AOA) and low affinity nitrifiers (LAN). This is governed by their differing growth responses, but is expressed as the deep ocean nitrification flux resulting for a given deep ocean ammonium reservoir, at a prescribed anoxic fraction of the ocean $f_{anoxic} = 0.32$, which corresponds to the point at which the growth responses are equal.

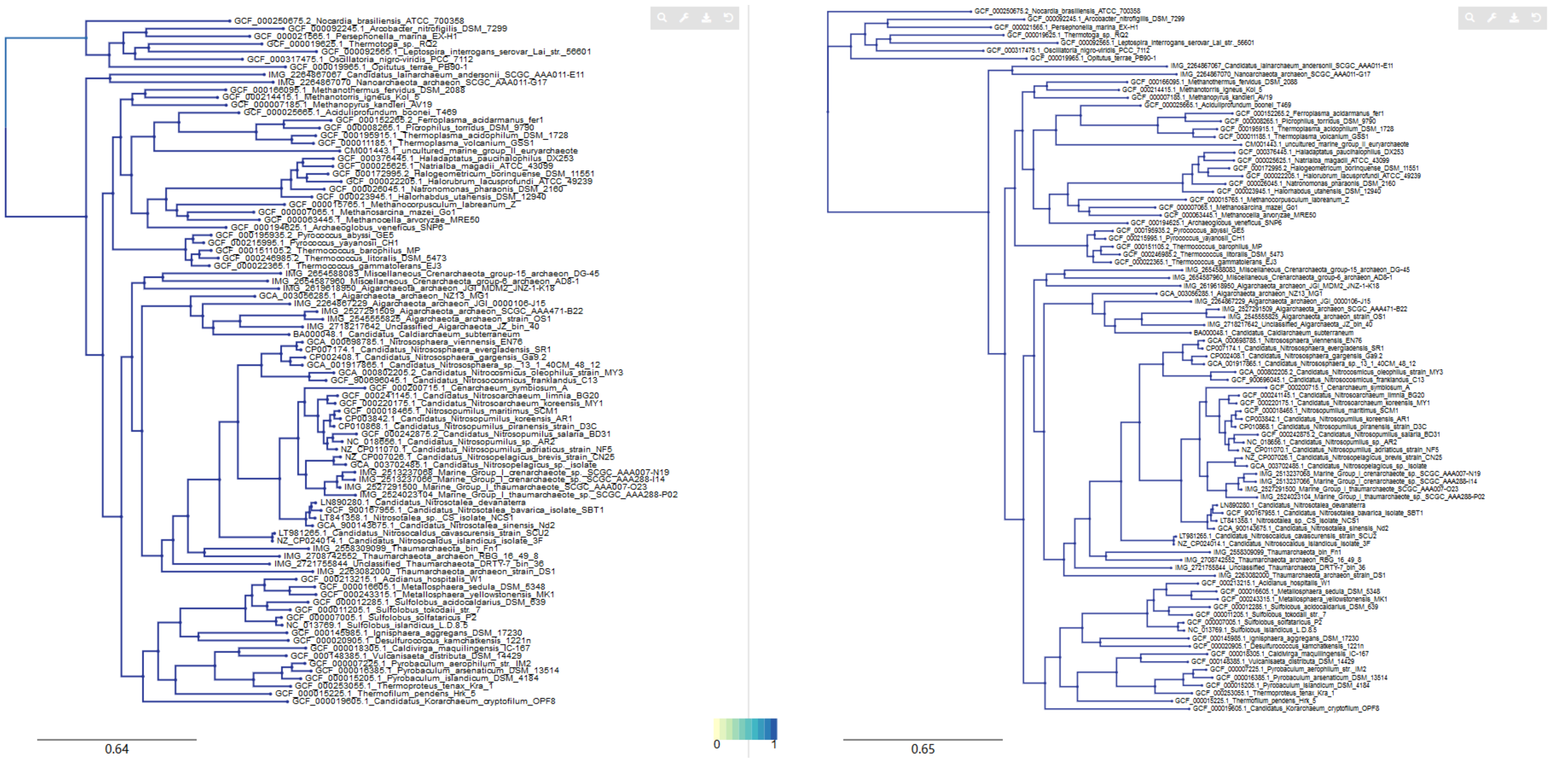

Fig. S4. Comparison of topology and branch lengths between phylogenetic trees constructed by RAxML (left) and IQ-Tree (right) with Phylo.io. The congruency index (Icong) is 4.594 (p-value=6.072e-35) for these two trees, calculated by icong (http://max2.ese.u-psud.fr/icong/index.help.html), indicating the tree topologies are significantly more congruent than expected by chance (de Vienne et al. 2007). The congruence between RAxML and IQ-Tree lies at not only the topologies but also the branch lengths (all shown in dark blue). The color bar at the bottom shows the consistence with dark blue indicating high similarity and white low similarity.

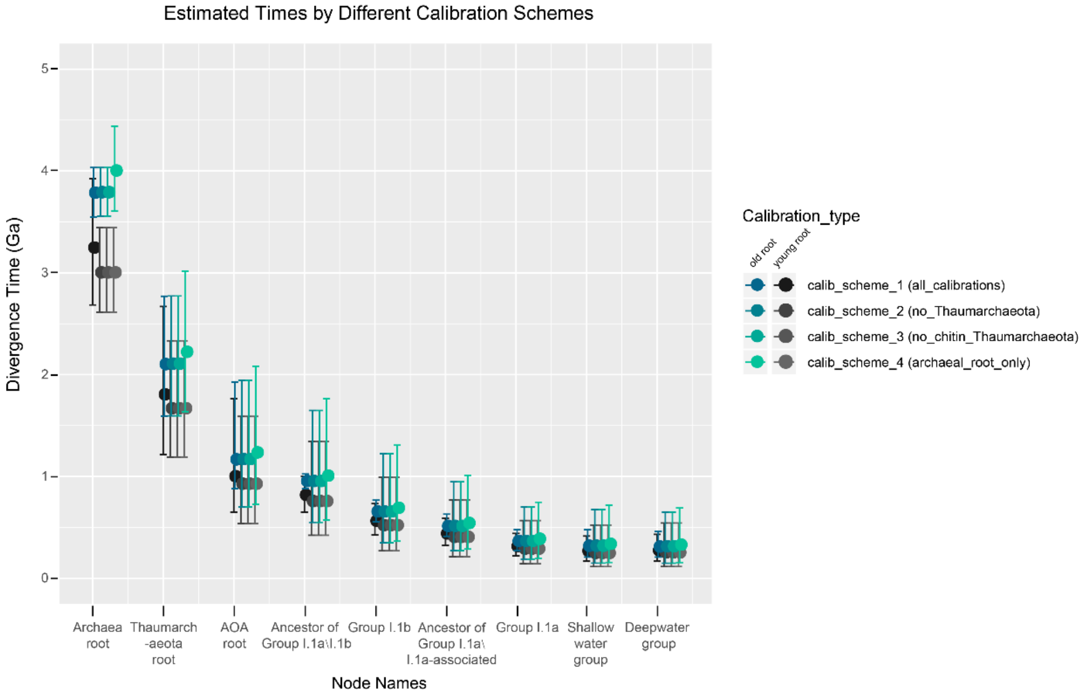

Fig. S5. Estimated node ages by different calibration schemes. A total of four calibration schemes were applied to estimate key node ages on the phylogenetic trees. Different schemes gave very similar results. It is also observed that the closer the estimated nodes were moving to the present, the more congruent the results were between the eight different scenarios, implying the increased reliability for estimated younger ages in this study.

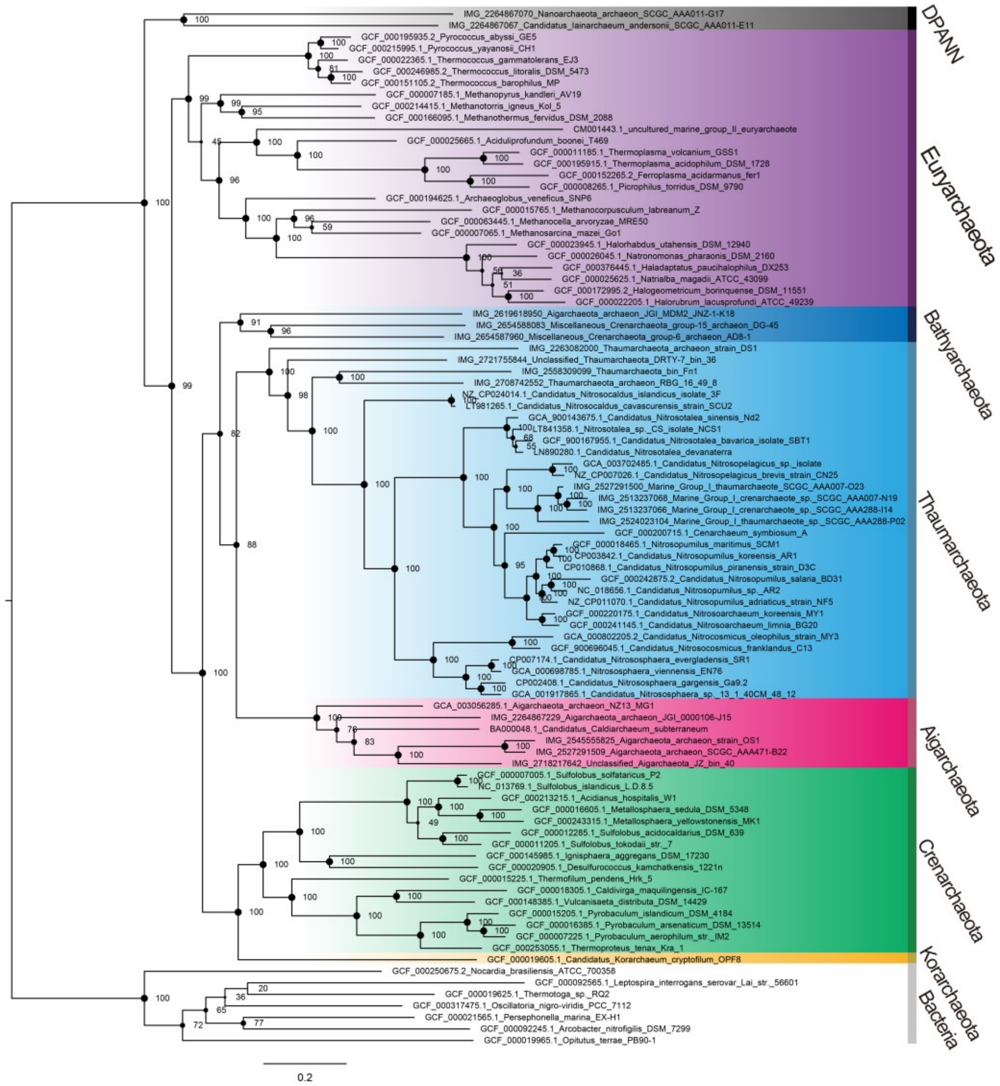

Fig. S6. Maximum likelihood (ML) phylogenomic tree constructed by RAxML v8.2.10 with bootstrap supporting values.

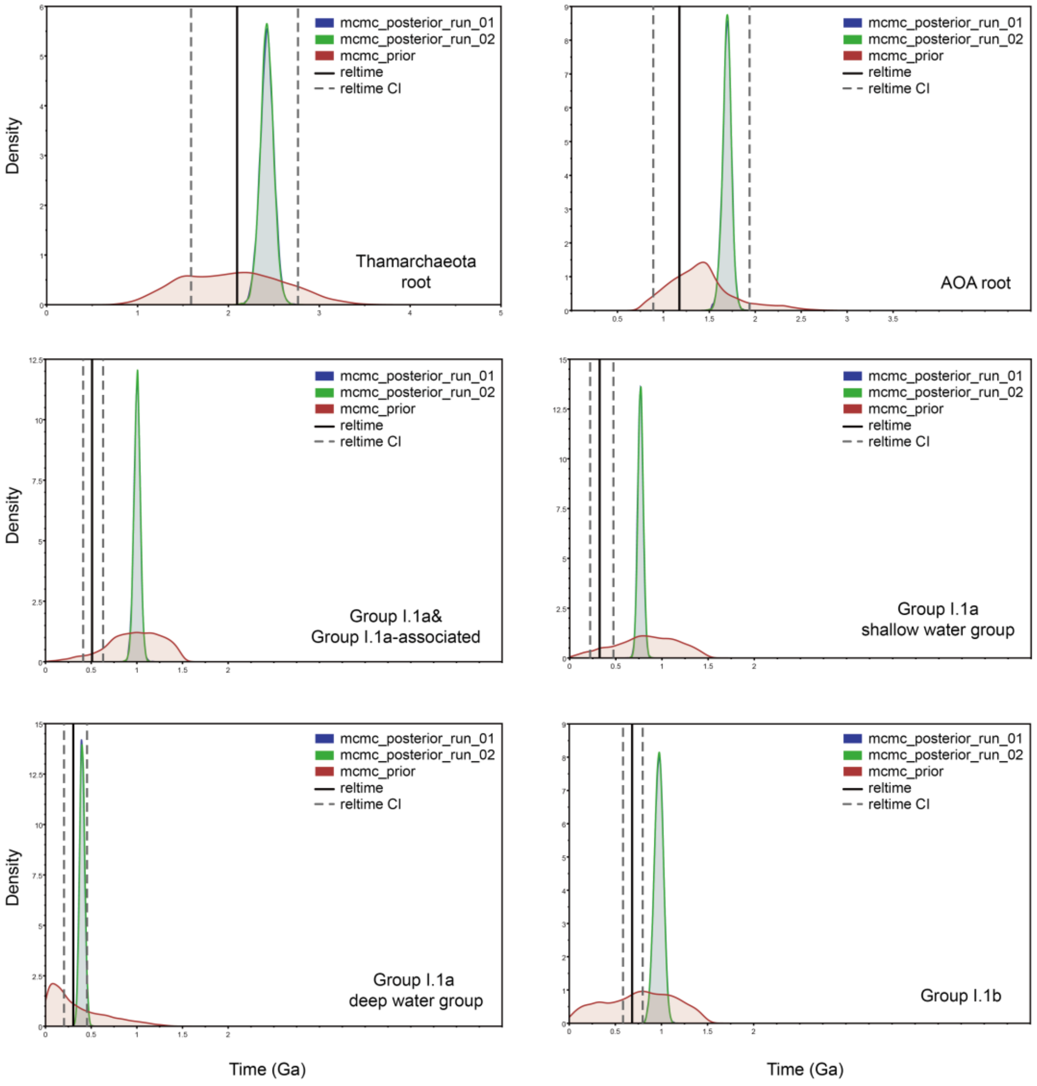

Fig. S7. Posterior (blue and green areas, almost overlapped here) and prior distributions (red area) estimated by two MCMCTree runs. Solid lines indicate node ages estimated by RelTime (with dash lines indicating the 94% CI).

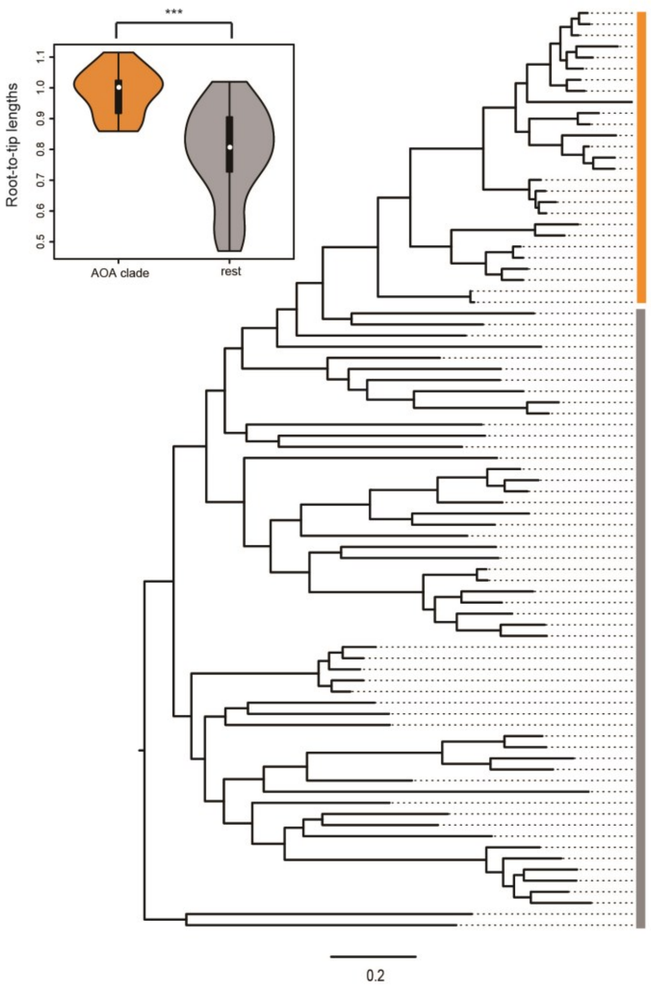

Fig. S8. Branch lengths comparison between AOA and other clades. Phylogenetic tree with branch lengths (main panel) and root-to-tip branch distances in violin plot (inserted panel) for AOA clade (orange) as compared with the rest of the tree (gray).

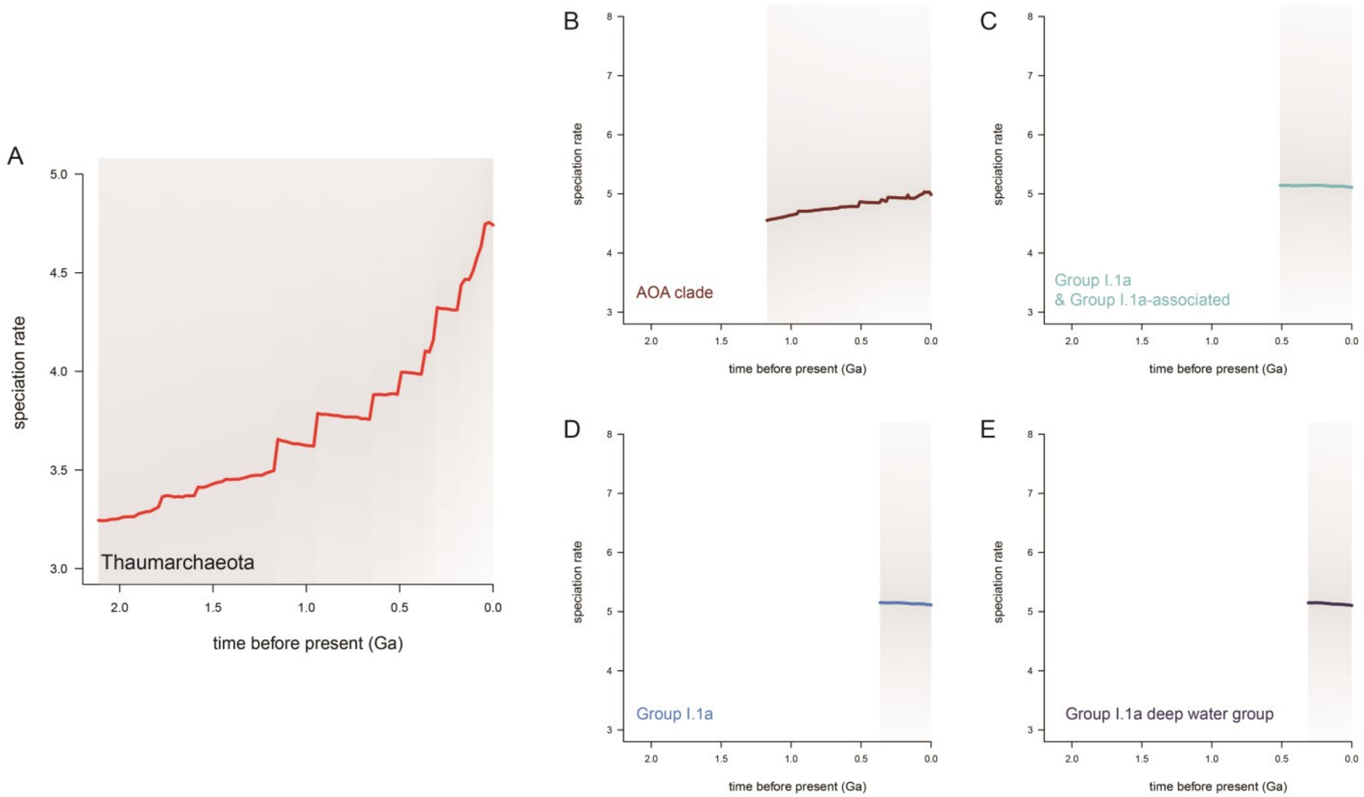

Fig. S9. Speciation rate plots estimated by BAMM for (A) the Thaumarchaeota phylum; (B) the AOA clade; (C) Group I.1a & Group I.1a-associated clades; (D) Group I.1a; (E) Group I.1a deep water group.

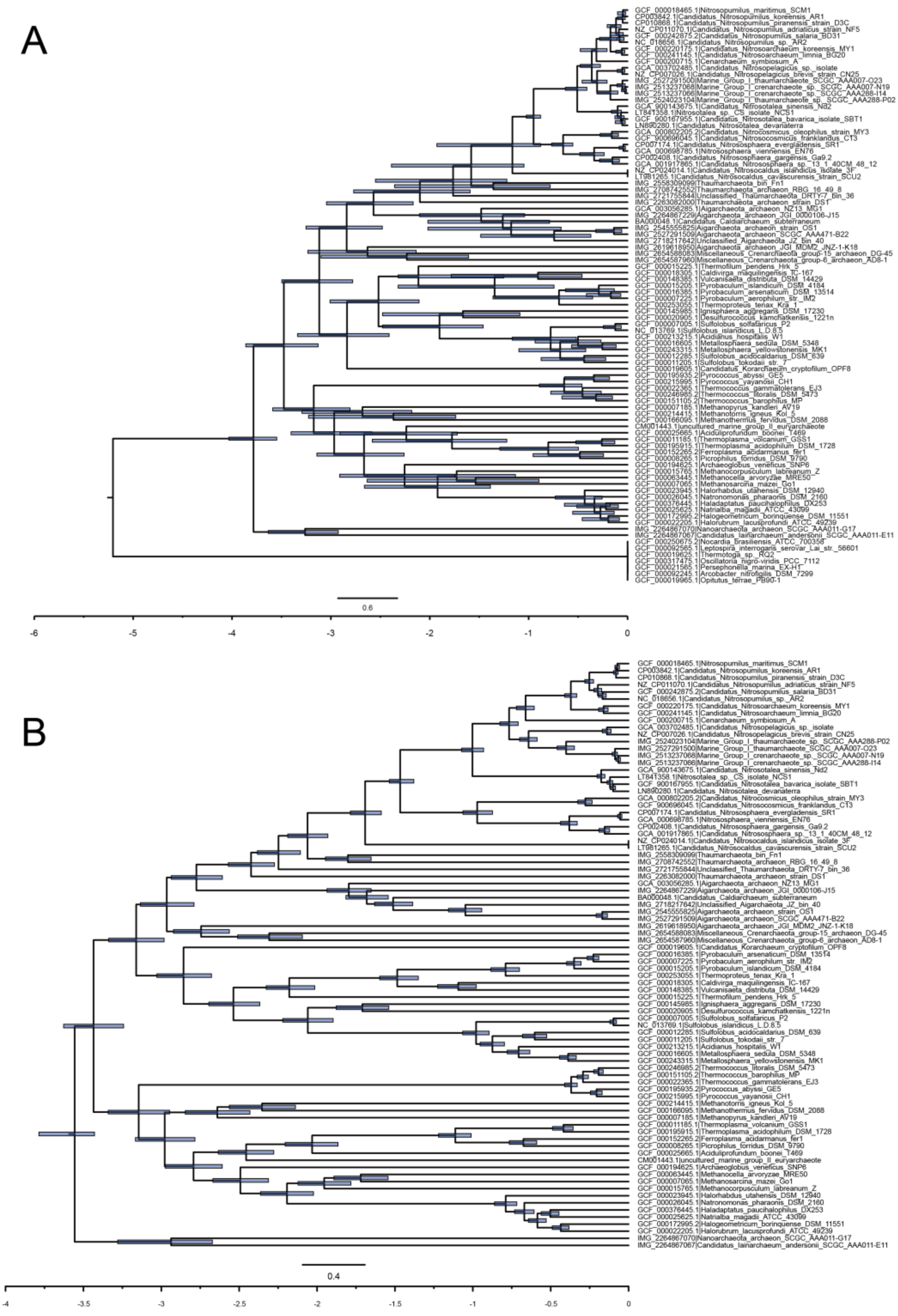

Fig. S10. The estimated timescale of phylogenomic tree (A) with 94% CI (blue bar) and bacterial as outgroups using RelTime and (B) with 95% CI (blue bar) using MCMCTree.

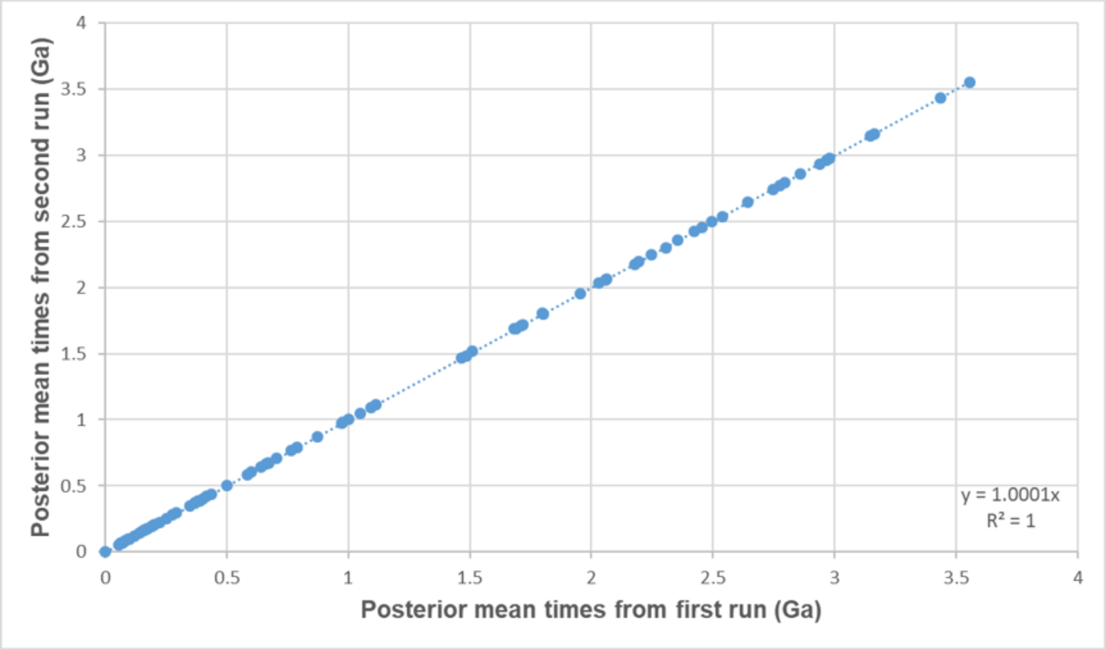

Fig. S11. Node ages between two runs by MCMCTree with a fitted function and R^2 indicating the relationship of variables and degree of consistence.

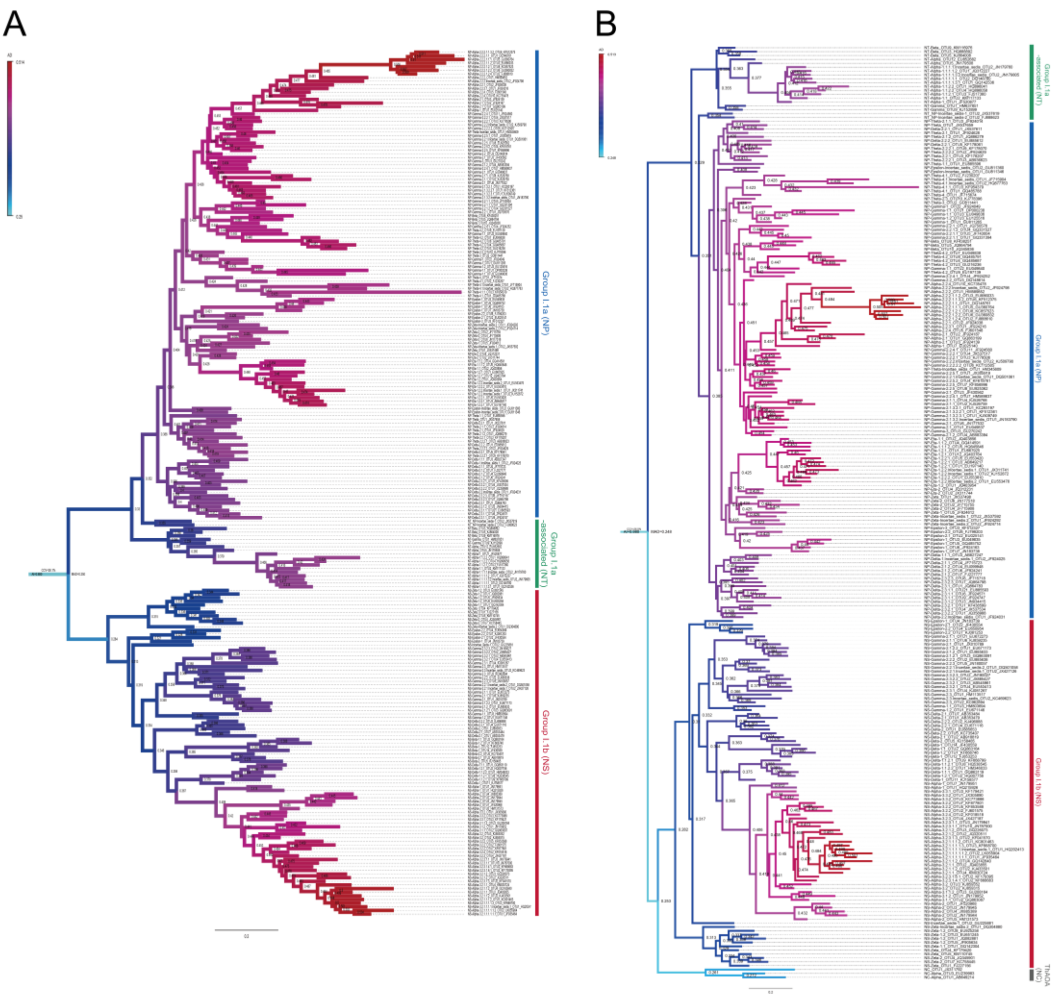

Fig. S12. (A) The AD (ancestor deviation) phylogenetic *amoA* tree without NC (also named ThAOA) group, with blue showing the low AD and red high AD. It is shown that the root was set between the NS group and the NP/NT group. (B) The AD (ancestor deviation) phylogenetic *amoA* tree with NC group. It is noted that the branches of NC group colored in light blue (low AD values) suggests its closest to the root of the tree so it was used as outgroups in this study.

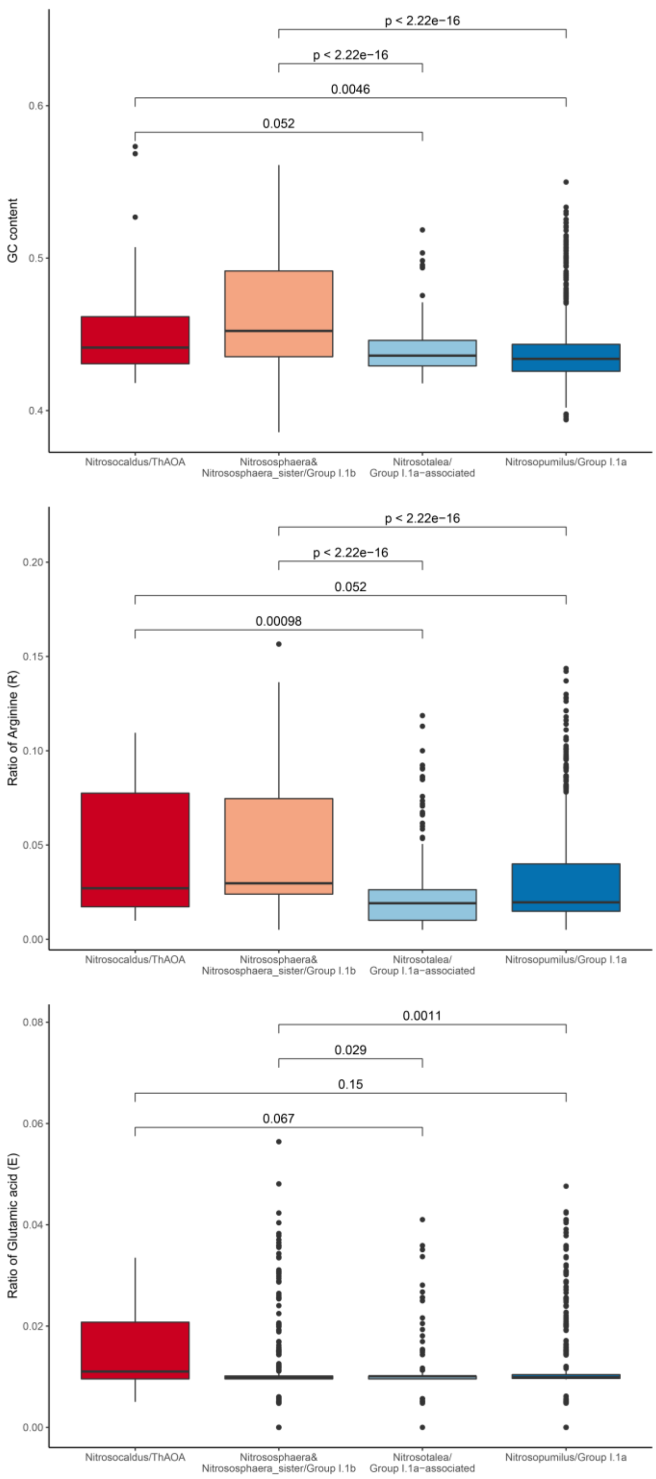

Fig. S13. Boxplots of GC contents and ratios of two charged amino acid possibly favoring thermostability (with p-values of two-sample Wilcoxon test between groups shown in the upper part of each panel). (A) GC contents among different clades of AOA. (B) The ratio of Arginine among different clades of AOA. (C) The ratio of Glutamic acid among different clades of AOA.

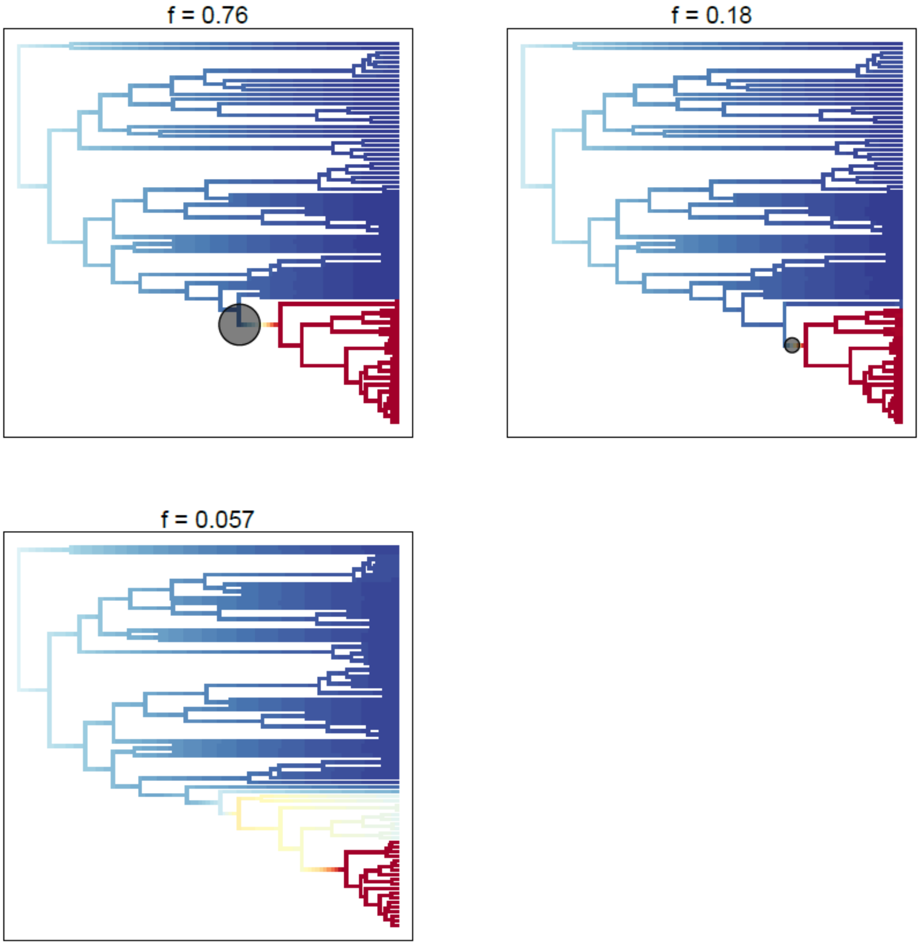

Fig. S14. Plots of the three most probable diversification rate shift configurations along with their individual contributions to the posterior distribution of all sampled BAMM models.

Table S1. Descriptions of the 90 genomes.

No.	Phylum/Class	Accession	Strain Name
**Bacteria**			
1	Actinobacteria	GCF_000250675.2	*Nocardia brasiliensis ATCC 700358*
2	Proteobacteria	GCF_000092245.1	*Arcobacter nitrofigilis DSM 7299*
3	Aquificae	GCF_000021565.1	*Persephonella marina EX-H1*
4	Chlamydiae/Verrucomicrobia	GCF_000019965.1	*Opitutus terrae PB90-1*
5	Thermotogae	GCF_000019625.1	*Thermotoga sp. RQ2*
6	Spirochaetes	GCF_000092565.1	*Leptospira interrogans serovar Lai str. 56601*
7	Cyanobacteria	GCF_000317475.1	*Oscillatoria nigro-viridis PCC 7112*
**Archaea**			
8	DPANN_group	IMG_2264867067	*Candidatus Iainarchaeum andersonii SCGC AAA011-E11*
9	DPANN_group	IMG_2264867070	*Nanoarchaeota archaeon SCGC AAA011-G17*
10	Euryarchaeota	GCF_000166095.1	*Methanothermus fervidus DSM 2088*
11	Euryarchaeota	GCF_000007185.1	*Methanopyrus kandleri AV19*
12	Euryarchaeota	GCF_000195935.2	*Pyrococcus abyssi GE5*
13	Euryarchaeota	GCF_000215995.1	*Pyrococcus yayanosii CH1*
14	Euryarchaeota	GCF_000151105.2	*Thermococcus barophilus MP*
15	Euryarchaeota	GCF_000022365.1	*Thermococcus gammatolerans EJ3*
16	Euryarchaeota	GCF_000246985.2	*Thermococcus litoralis DSM 5473*
17	Euryarchaeota	GCF_000025665.1	*Aciduliprofundum boonei T469*
18	Euryarchaeota	GCF_000152265.2	*Ferroplasma acidarmanus fer1*
19	Euryarchaeota	GCF_000008265.1	*Picrophilus torridus DSM 9790*
20	Euryarchaeota	GCF_000195915.1	*Thermoplasma acidophilum DSM 1728*
21	Euryarchaeota	GCF_000011185.1	*Thermoplasma volcanium GSS1*
22	Euryarchaeota	GCF_000376445.1	*Haladaptatus paucihalophilus DX253*
23	Euryarchaeota	GCF_000172995.2	*Halogeometricum borinquense DSM 11551*
24	Euryarchaeota	GCF_000023945.1	*Halorhabdus utahensis DSM 12940*
25	Euryarchaeota	GCF_000022205.1	*Halorubrum lacusprofundi ATCC 49239*
26	Euryarchaeota	GCF_000025625.1	*Natrialba magadii ATCC 43099*
27	Euryarchaeota	GCF_000026045.1	*Natronomonas pharaonis DSM 2160*
28	Euryarchaeota	GCF_000015765.1	*Methanocorpusculum labreanum Z*
29	Euryarchaeota	GCF_000007065.1	*Methanosarcina mazei Go1*
30	Euryarchaeota	GCF_000063445.1	*Methanocella arvoryzae MRE50*
31	Euryarchaeota	GCA_000246735.1	*uncultured marine group II euryarchaeote*
32	Euryarchaeota	GCF_000194625.1	*Archaeoglobus veneficus SNP6*
33	Euryarchaeota	GCF_000214415.1	*Methanotorris igneus Kol 5*
34	Crenarchaeota	GCF_000213215.1	*Acidianus hospitalis W1*
35	Crenarchaeota	GCF_000016605.1	*Metallosphaera sedula DSM 5348*
36	Crenarchaeota	GCF_000243315.1	*Metallosphaera yellowstonensis MK1*
37	Crenarchaeota	GCF_000012285.1	*Sulfolobus acidocaldarius DSM 639*
38	Crenarchaeota	GCF_000007005.1	*Sulfolobus solfataricus P2*

39	Crenarchaeota	GCA_000024305.1	*Sulfolobus islandicus L.D.8.5*
40	Crenarchaeota	GCF_000011205.1	*Sulfolobus tokodaii str. 7*
41	Crenarchaeota	GCF_000018305.1	*Caldivirga maquilingensis IC-167*
42	Crenarchaeota	GCF_000007225.1	*Pyrobaculum aerophilum str. IM2*
43	Crenarchaeota	GCF_000016385.1	*Pyrobaculum arsenaticum DSM 13514*
44	Crenarchaeota	GCF_000015205.1	*Pyrobaculum islandicum DSM 4184*
45	Crenarchaeota	GCF_000015225.1	*Thermofilum pendens Hrk 5*
46	Crenarchaeota	GCF_000253055.1	*Thermoproteus tenax Kra 1*
47	Crenarchaeota	GCF_000148385.1	*Vulcanisaeta distributa DSM 14429*
48	Crenarchaeota	GCF_000145985.1	*Ignisphaera aggregans DSM 17230*
49	Crenarchaeota	GCF_000020905.1	*Desulfurococcus kamchatkensis 1221n*
50	Korarchaeota	GCF_000019605.1	*Candidatus Korarchaeum cryptofilum OPF8*
51	Bathyarchaeota	IMG_2654588083	*Miscellaneous Crenarchaeota group-15 archaeon DG-45*
52	Bathyarchaeota	IMG_2654587960	*Miscellaneous Crenarchaeota group-6 archaeon AD8-1*
53	Aigarchaeota	GCA_003056285.1	*Aigarchaeota archaeon NZ13 MG1*
54	Aigarchaeota	IMG_2264867229	*Aigarchaeota archaeon JGI 0000106-J15*
55	Aigarchaeota	IMG_2527291509	*Aigarchaeota archaeon SCGC AAA471-B22*
56	Aigarchaeota	IMG_2545555825	*Aigarchaeota archaeon strain OS1*
57	Aigarchaeota	IMG_2718217642	*Unclassified Aigarchaeota JZ bin 40*
58	Aigarchaeota	IMG_2619618950	*Aigarchaeota archaeon JGI MDM2 JNZ-1-K18*
59	Aigarchaeota	GCF_000270325.1	*Candidatus Caldiarchaeum subterraneum*
60	Thaumarchaeota	GCA_000698785.1	*Nitrososphaera viennensis EN76*
61	Thaumarchaeota	GCF_000303155.1	*Candidatus Nitrososphaera gargensis Ga9.2*
62	Thaumarchaeota	GCF_000730285.1	*Candidatus Nitrososphaera evergladensis SR1*
63	Thaumarchaeota	GCF_000200715.1	*Cenarchaeum symbiosum A*
64	Thaumarchaeota	GCF_000241145.1	*Candidatus Nitrosoarchaeum limnia BG20*
65	Thaumarchaeota	GCF_000220175.1	*Candidatus Nitrosoarchaeum koreensis MY1*
66	Thaumarchaeota	GCF_000018465.1	*Nitrosopumilus maritimus SCM1*
67	Thaumarchaeota	GCF_000242875.2	*Candidatus Nitrosopumilus salaria BD31*
68	Thaumarchaeota	GCF_900248165.1	*Candidatus Nitrosocaldus cavascurensis strain SCU2*
69	Thaumarchaeota	GCF_002906215.1	*Candidatus Nitrosocaldus islandicus isolate 3F*
70	Thaumarchaeota	GCF_900065925.1	*Candidatus Nitrosotalea devanaterra*
71	Thaumarchaeota	GCF_900167955.1	*Candidatus Nitrosotalea bavarica isolate SBT1*
72	Thaumarchaeota	GCA_900143675.1	*Candidatus Nitrosotalea sinensis Nd2*
73	Thaumarchaeota	GCA_900177045.1	*Nitrosotalea sp. CS isolate NCS1*
74	Thaumarchaeota	GCF_000299365.1	*Candidatus Nitrosopumilus koreensis AR1*
75	Thaumarchaeota	GCF_000299395.1	*Candidatus Nitrosopumilus sp. AR2*
76	Thaumarchaeota	GCF_000956175.1	*Candidatus Nitrosopumilus adriaticus strain NF5*
77	Thaumarchaeota	GCF_000875775.1	*Candidatus Nitrosopumilus piranensis strain D3C*

78	Thaumarchaeota	GCA_001917865.1	*Candidatus Nitrososphaera sp. 13 1 40CM 48 12*
79	Thaumarchaeota	GCA_000802205.2	*Candidatus Nitrosocosmicus oleophilus strain MY3*
80	Thaumarchaeota	GCF_900696045.1	*Candidatus Nitrosocosmicus franklandus C13*
81	Thaumarchaeota	GCF_000812185.1	*Candidatus Nitrosopelagicus brevis strain CN25*
82	Thaumarchaeota	GCA_003702485.1	*Candidatus Nitrosopelagicus sp. Isolate GoM MAG1*
83	Thaumarchaeota	IMG_2513237068	*Marine Group I crenarchaeote sp. SCGC AAA007-N19*
84	Thaumarchaeota	IMG_2527291500	*Marine Group I thaumarchaeote SCGC AAA007-O23*
85	Thaumarchaeota	IMG_2513237066	*Marine Group I crenarchaeote sp. SCGC AAA288-I14*
86	Thaumarchaeota	IMG_2524023104	*Marine Group I thaumarchaeote sp. SCGC AAA288-P02*
87	Thaumarchaeota	IMG_2558309099	*Thaumarchaeota bin Fn1*
88	Thaumarchaeota	IMG_2263082000	*Thaumarchaeota archaeon strain DS1*
89	Thaumarchaeota	IMG_2708742552	*Thaumarchaeota archaeon RBG 16 49 8*
90	Thaumarchaeota	IMG_2721755844	*Unclassified Thaumarchaeota DRTY-7 bin 36*

Table S2. List of the seventy marker proteins.

Accession	Protein Name	Species
**BAJ47301.1**	prolyl-tRNA synthetase	*Candidatus Caldiarchaeum subterraneum*
**BAJ47360.1**	histidyl-tRNA synthetase	*Candidatus Caldiarchaeum subterraneum*
**BAJ47583.1**	signal recognition particle receptor	*Candidatus Caldiarchaeum subterraneum*
**BAJ48087.1**	hydrogenase maturation protein HypF, partial	*Candidatus Caldiarchaeum subterraneum*
**BAJ48095.1**	hydrogenase expression/formation protein HypD	*Candidatus Caldiarchaeum subterraneum*
**BAJ48371.1**	5'-nucleotidase SurE	*Candidatus Caldiarchaeum subterraneum*
**BAJ48542.1**	conserved hypothetical protein	*Candidatus Caldiarchaeum subterraneum*
**BAJ48802.1**	molybdenum cofactor biosynthesis protein C	*Candidatus Caldiarchaeum subterraneum*
**YP_001581379.1**	homoserine kinase	*Nitrosopumilus maritimus SCM1*
**YP_001581437.1**	glutamyl-tRNA(Gln) amidotransferase subunit E	*Nitrosopumilus maritimus SCM1*
**YP_001581440.1**	50S ribosomal protein L2	*Nitrosopumilus maritimus SCM1*
**YP_001581625.1**	peptidase M50	*Nitrosopumilus maritimus SCM1*
**YP_001581650.1**	30S ribosomal protein S2	*Nitrosopumilus maritimus SCM1*
**YP_001581658.1**	30S ribosomal protein S4	*Nitrosopumilus maritimus SCM1*
**YP_001581659.1**	30S ribosomal protein S13	*Nitrosopumilus maritimus SCM1*
**YP_001581688.1**	30S ribosomal protein S12	*Nitrosopumilus maritimus SCM1*
**YP_001581689.1**	30S ribosomal protein S7	*Nitrosopumilus maritimus SCM1*
**YP_001581715.1**	acidic ribosomal protein P0	*Nitrosopumilus maritimus SCM1*
**YP_001581716.1**	50S ribosomal protein L1	*Nitrosopumilus maritimus SCM1*
**YP_001581719.1**	50S ribosomal protein L11	*Nitrosopumilus maritimus SCM1*
**YP_001581732.1**	50S ribosomal protein L18	*Nitrosopumilus maritimus SCM1*
**YP_001581733.1**	30S ribosomal protein S5	*Nitrosopumilus maritimus SCM1*
**YP_001581734.1**	50S ribosomal protein L30	*Nitrosopumilus maritimus SCM1*
**YP_001581735.1**	50S ribosomal protein L15	*Nitrosopumilus maritimus SCM1*
**YP_001581759.1**	30S ribosomal protein S9	*Nitrosopumilus maritimus SCM1*
**YP_001581760.1**	50S ribosomal protein L13	*Nitrosopumilus maritimus SCM1*
**YP_001581766.1**	exosome complex exonuclease 1	*Nitrosopumilus maritimus SCM1*
**YP_001581783.1**	50S ribosomal protein L10e	*Nitrosopumilus maritimus SCM1*
**YP_001581824.1**	glutamate-1-semialdehyde-2,1-aminomutase	*Nitrosopumilus maritimus SCM1*
**YP_001581834.1**	phosphoglycerate kinase	*Nitrosopumilus maritimus SCM1*
**YP_001581865.1**	GTP-binding signal recognition particle	*Nitrosopumilus maritimus SCM1*

**YP_001581916.1**	phosphoribosylformylglycinamidine cyclo-ligase	*Nitrosopumilus maritimus SCM1*
**YP_001581960.1**	LPPG:FO 2-phospho-L-lactate transferase	*Nitrosopumilus maritimus SCM1*
**YP_001582030.1**	homoserine dehydrogenase	*Nitrosopumilus maritimus SCM1*
**YP_001582114.1**	phosphoribosylformylglycinamidine synthase II	*Nitrosopumilus maritimus SCM1*
**YP_001582115.1**	class II glutamine amidotransferase	*Nitrosopumilus maritimus SCM1*
**YP_001582128.1**	50S ribosomal protein L6	*Nitrosopumilus maritimus SCM1*
**YP_001582129.1**	30S ribosomal protein S8	*Nitrosopumilus maritimus SCM1*
**YP_001582131.1**	50S ribosomal protein L5	*Nitrosopumilus maritimus SCM1*
**YP_001582133.1**	KOW domain-containing protein	*Nitrosopumilus maritimus SCM1*
**YP_001582134.1**	50S ribosomal protein L14	*Nitrosopumilus maritimus SCM1*
**YP_001582135.1**	30S ribosomal protein S17	*Nitrosopumilus maritimus SCM1*
**YP_001582137.1**	50S ribosomal protein L29	*Nitrosopumilus maritimus SCM1*
**YP_001582138.1**	30S ribosomal protein S3	*Nitrosopumilus maritimus SCM1*
**YP_001582139.1**	50S ribosomal protein L22	*Nitrosopumilus maritimus SCM1*
**YP_001582140.1**	30S ribosomal protein S19	*Nitrosopumilus maritimus SCM1*
**YP_001582141.1**	50S ribosomal protein L25	*Nitrosopumilus maritimus SCM1*
**YP_001582142.1**	50S ribosomal protein L4P	*Nitrosopumilus maritimus SCM1*
**YP_001582143.1**	50S ribosomal protein L3P	*Nitrosopumilus maritimus SCM1*
**YP_001582202.1**	glutamyl-tRNA(Gln) amidotransferase subunit B	*Nitrosopumilus maritimus SCM1*
**YP_001582367.1**	30S ribosomal protein S10	*Nitrosopumilus maritimus SCM1*
**YP_001582595.1**	MiaB-like tRNA modifying enzyme	*Nitrosopumilus maritimus SCM1*
**YP_001582620.1**	argininosuccinate synthase	*Nitrosopumilus maritimus SCM1*
**YP_001582632.1**	cytidyltransferase-like protein	*Nitrosopumilus maritimus SCM1*
**YP_001582784.1**	30S ribosomal protein S11	*Nitrosopumilus maritimus SCM1*
**YP_001582789.1**	adenylosuccinate lyase	*Nitrosopumilus maritimus SCM1*
**YP_001582813.1**	pyridoxal biosynthesis lyase PdxS	*Nitrosopumilus maritimus SCM1*
**YP_001582823.1**	phenylalanyl-tRNA synthetase subunit alpha	*Nitrosopumilus maritimus SCM1*
**YP_001582842.1**	30S ribosomal protein S15	*Nitrosopumilus maritimus SCM1*
**YP_001582864.1**	RdgB/HAM1 family non-canonical purine NTP pyrophosphatase	*Nitrosopumilus maritimus SCM1*
**YP_001582867.1**	metalloendopeptidase glycoprotease family	*Nitrosopumilus maritimus SCM1*
**YP_001582870.1**	tRNA-guanine transglycosylase	*Nitrosopumilus maritimus SCM1*
**YP_001582873.1**	beta-lactamase domain-containing protein	*Nitrosopumilus maritimus SCM1*
**YP_001582931.1**	UbiD family decarboxylase	*Nitrosopumilus maritimus SCM1*
**YP_001582939.1**	GTP1/OBG protein	*Nitrosopumilus maritimus SCM1*
**YP_001583020.1**	aspartate carbamoyltransferase	*Nitrosopumilus maritimus SCM1*
**YP_001583039.1**	uridylate kinase	*Nitrosopumilus maritimus SCM1*
**YP_001583055.1**	phosphopantothenoylcysteine decarboxylase/phosphopantothenate--cysteine ligase	*Nitrosopumilus maritimus SCM1*

**YP_001583088.1**	aspartate kinase	*Nitrosopumilus maritimus SCM1*
**YP_876283.1**	porphobilinogen deaminase	*Cenarchaeum symbiosum A*

Table S3. The sensitive test on calibration points by adding or subtracting 200 million years.

Constraint Type	Adding 200 million years		Subtracting 200 million years	
	RMSE[a]	P-value[b]	RMSE	P-value
Oxygen age constraints	0.0358	0.8789	0.0755	0.7387
Chitin age constraints	0	1	0	1
HGT constraint from Viridiplantae to Thaumarchaeota	0.0019	0.9934	0	1

[a] RMSE: Root Mean Square Error compared with the original results. The less this number is, the less difference there is from the original results.

[b] P-value: The two-tailed t-test between shifting-constraint results and the original one. The bigger this number is, the less difference there is from the original results.

Table S4. Comparison of the MCMCTree results between this study and Ren *et al.*

Node name	Age	Lower bound	Upper bound	Node name	Age	Lower bound	Upper bound
**This study**				**Ren et al.**			
Thaumarchaeota root	2.424	2.272	2.568	Node 1	2.631	2.518	2.765
AOA clade root	1.692	1.590	1.787	Node 2	2.165	2.06	2.285
Ancestor of group I.1a	0.767	0.711	0.827	Node 3	1.017	0.959	1.084
Deep water group	0.401	0.345	0.456	Node 4	0.643	0.588	0.701

Table S5. Seven calibration nodes and their time ranges.

Node Calibration Type	Clade Name	Calibration time (Ga)		References
		Maximum	Minimum	
Archaea Root Constraint	Archaea Root	4.38[a]; 3.8[b]	3.46[a]; 2.7[b]	[a](Valley et al. 2014),(Ueno et al. 2009),(Wolfe and Fournier 2018); [b](Blank 2009a),(Blank 2011)
Oxygen Age Constraint	*Thermoproteales*	2.32	\	(Blank 2009a),(Blank 2011)
Oxygen Age Constraint	*Sulfolobales*	2.32	\	(Blank 2009a),(Blank 2011)
Oxygen Age Constraint	*Thermoplasma*	2.32	\	(Blank 2009a),(Blank 2011)
Chitin Age Constraint	*Thermococcales*	1.579	\	(Parfrey et al. 2011), (Eme et al. 2014)
Chitin Age Constraint	*Halobacteriales*	1.579	\	(Parfrey et al. 2011), (Eme et al. 2014)
HGT from Viridiplantae to Thaumarchaeota	*Nitrososphaera-Nitrosopumilus*	1.487	0.75	(Douzery et al. 2004),(Petitjean et al. 2012),(Parfrey et al. 2011)

[a] old root calibration

[b] young root calibration

Table S6. Best substitution models determined for 70 marker genes suggested by PartifionFinder v2.1.1.

Best Model	RAxML Best Model	Partition Names	Sequence Site Positions
LG+I+G	LG	BAJ47360,YP_001582939,BAJ47301,YP_001581440	297-474,10981-11182,1-296,2323-2501
LG+I+G	LG	BAJ47583,YP_001581834,YP_001582870,YP_001581916	475-647,4854-5093,10339-10561,5395-5572
LG+G	LG	BAJ48087,YP_001582632	648-1112,8982-9061
LG+G	LG	BAJ48095,YP_001582873	1113-1386,10562-10757
LG+G	LG	BAJ48371	1387-1497
LG+I+G	LG	YP_001581760,BAJ48542	4236-4314,1498-1721
LG+I+G	LG	BAJ48802,YP_001582823,YP_001582030	1722-1820,9675-9896,5793-5977
LG+G	LG	YP_001581379	1821-1969
LG+G	LG	YP_001581437	1970-2322
BLOSUM62+G	BLOSUM62	YP_001581625	2502-2580
LG+I+G	LG	YP_001581650,YP_001582138	2581-2725,7280-7427
LG+I+G	LG	YP_001581716,YP_001581735,YP_001581658,YP_001582135,YP_001583039	3414-3540,4074-4144,2726-2827,7145-7231,11392-11517
LG+I+G	LG	YP_001581759,YP_001581659	4145-4235,2828-2933
LG+G	LG	YP_001581688,YP_001582784	2934-3068,9062-9183
LG+G	LG	YP_001581689,YP_001581783	3069-3234,4521-4636
LG+G	LG	YP_001581715	3235-3413
LG+G	LG	YP_001581719	3541-3684
LG+G	LG	YP_001582142,YP_001581732,YP_001581734	7717-7881,3685-3807,3987-4073
LG+G	LG	YP_001581733	3808-3986
LG+I+G	LG	YP_001581865,YP_001581766,YP_001582129	5094-5394,4315-4520,6702-6813
BLOSUM62+I+G	BLOSUM62	YP_001581824	4637-4853
LG+I+G	LG	YP_001581960,YP_001582595	5573-5792,8504-8669
LG+I+G	LG	YP_001582114	5978-6412
BLOSUM62+I+G	BLOSUM62	YP_001582115	6413-6595
LG+I+G	LG	YP_001582133,YP_001582128	6951-7025,6596-6701
LG+G	LG	YP_001582131	6814-6950
LG+I+G	LG	YP_001582134	7026-7144

LG+I+G	LG	YP_001582842,YP_001582137	9897-10019,7232-7279
LG+I+G	LG	YP_001582139,YP_001582143	7428-7534,7882-8092
LG+I+G	LG	YP_001582140,YP_001582367	7535-7640,8414-8503
LG+G	LG	YP_001582141	7641-7716
LG+I+G	LG	YP_001582202,YP_001583020	8093-8413,11183-11391
LG+I+G	LG	YP_001582620	8670-8981
BLOSUM62+I+G	BLOSUM62	YP_001582789	9184-9411
LG+I+G	LG	YP_001582813	9412-9674
LG+I+G	LG	YP_001582864	10020-10115
LG+I+G	LG	YP_001582867	10116-10338
LG+G	LG	YP_001582931	10758-10980
LG+G	LG	YP_001583055	11518-11747
BLOSUM62+I+G	BLOSUM62	YP_001583088	11748-11912
LG+I+G	LG	YP_876283	11913-12082

Table S7. The completeness, contamination ratios and types of genomes used in this study.

Accession	Type	Strain Name	Completeness	Contamination
GCF_000007005.1	Reference	*Sulfolobus solfataricus P2*	100.000	0.000
GCF_000007065.1	Reference	*Methanosarcina mazei Go1*	100.000	0.654
GCF_000007185.1	Reference	*Methanopyrus kandleri AV19*	96.738	1.600
GCF_000007225.1	Reference	*Pyrobaculum aerophilum str. IM2*	100.000	0.735
GCF_000008265.1	Reference	*Picrophilus torridus DSM 9790*	99.593	0.000
GCF_000011185.1	Reference	*Thermoplasma volcanium GSS1*	97.967	0.000
GCF_000011205.1	Reference	*Sulfolobus tokodaii str. 7*	100.000	0.000
GCF_000012285.1	Reference	*Sulfolobus acidocaldarius DSM 639*	99.405	0.000
GCF_000015205.1	Reference	*Pyrobaculum islandicum DSM 4184*	100.000	0.000
GCF_000015225.1	Reference	*Thermofilum pendens Hrk 5*	98.529	0.735
GCF_000015765.1	Reference	*Methanocorpusculum labreanum Z*	99.536	0.000
GCF_000016385.1	Reference	*Pyrobaculum arsenaticum DSM 13514*	100.000	0.000
GCF_000016605.1	Reference	*Metallosphaera sedula DSM 5348*	100.000	0.000
GCF_000018305.1	Reference	*Caldivirga maquilingensis IC-167*	99.265	0.000
GCF_000018465.1	Reference	*Nitrosopumilus maritimus SCM1*	100.000	0.971
GCF_000019605.1	Reference	*Candidatus Korarchaeum cryptofilum OPF8*	93.386	2.804
GCF_000019625.1	Reference	*Thermotoga sp. RQ2*	100.000	1.786
GCF_000019965.1	Reference	*Opitutus terrae PB90-1*	99.315	3.425

GCF_000020905.1	Reference	*Desulfurococcus kamchatkensis 1221n*	100.000	0.000
GCF_000021565.1	Reference	*Persephonella marina EX-H1*	99.797	1.829
GCF_000022205.1	Reference	*Halorubrum lacusprofundi ATCC 49239*	99.756	0.000
GCF_000022365.1	Reference	*Thermococcus gammatolerans EJ3*	100.000	0.000
GCF_000023945.1	Reference	*Halorhabdus utahensis DSM 12940*	98.253	0.867
GCF_000025625.1	Reference	*Natrialba magadii ATCC 43099*	99.173	0.000
GCF_000025665.1	Reference	*Aciduliprofundum boonei T469*	100.000	0.000
GCF_000026045.1	Reference	*Natronomonas pharaonis DSM 2160*	99.291	1.277
GCF_000063445.1	Reference	*Methanocella arvoryzae MRE50*	100.000	0.000
GCF_000092245.1	Reference	*Arcobacter nitrofigilis DSM 7299*	99.593	2.981
GCF_000092565.1	Reference	*Leptospira interrogans serovar Lai str. 56601*	96.471	0.000
GCF_000145985.1	Reference	*Ignisphaera aggregans DSM 17230*	100.000	0.000
GCF_000148385.1	Reference	*Vulcanisaeta distributa DSM 14429*	99.265	0.735
GCF_000151105.2	Reference	*Thermococcus barophilus MP*	99.505	0.000
GCF_000152265.2	Reference	*Ferroplasma acidarmanus fer1*	98.738	0.813
GCF_000166095.1	Reference	*Methanothermus fervidus DSM 2088*	100.000	0.000
GCF_000172995.2	Reference	*Halogeometricum borinquense DSM 11551*	99.924	0.048
GCF_000194625.1	Reference	*Archaeoglobus veneficus SNP6*	99.346	0.000
GCF_000195915.1	Reference	*Thermoplasma acidophilum DSM 1728*	97.518	0.000

GCF_000195935.2	Reference	*Pyrococcus abyssi GE5*	100.000	0.000
GCF_000200715.1	Reference	*Cenarchaeum symbiosum A*	99.029	0.000
GCF_000213215.1	Reference	*Acidianus hospitalis W1*	99.405	0.000
GCF_000214415.1	Reference	*Methanotorris igneus Kol 5*	99.524	0.000
GCF_000215995.1	Reference	*Pyrococcus yayanosii CH1*	100.000	0.000
GCF_000220175.1	Reference	*Candidatus Nitrosoarchaeum koreensis MY1*	100.000	0.000
GCF_000241145.1	Reference	*Candidatus Nitrosoarchaeum limnia BG20*	99.029	5.825
GCF_000242875.2	Reference	*Candidatus Nitrosopumilus salaria BD31*	92.395	1.942
GCF_000243315.1	Reference	*Metallosphaera yellowstonensis MK1*	100.000	0.000
GCF_000246985.2	Reference	*Thermococcus litoralis DSM 5473*	99.505	0.495
GCF_000250675.2	Reference	*Nocardia brasiliensis ATCC 700358*	99.373	4.840
GCF_000253055.1	Reference	*Thermoproteus tenax Kra 1*	100.000	0.735
GCF_000270325.1	Reference	*Candidatus Caldiarchaeum subterraneum*	98.058	0.000
GCF_000299365.1	Reference	*Candidatus Nitrosopumilus koreensis AR1*	94.660	0.000
GCF_000299395.1	Reference	*Candidatus Nitrosopumilus sp. AR2*	97.087	0.000
GCF_000303155.1	Reference	*Candidatus Nitrososphaera gargensis Ga9.2*	100.000	2.913
GCF_000317475.1	Reference	*Oscillatoria nigro-viridis PCC 7112*	99.782	0.218

GCF_000376445.1	Reference	*Haladaptatus paucihalophilus DX253*	99.078	0.851
GCF_000730285.1	Reference	*Candidatus Nitrososphaera evergladensis SR1*	100.000	2.913
GCF_000812185.1	Reference	*Candidatus Nitrosopelagicus brevis strain CN25*	99.515	0.000
GCF_000875775.1	Reference	*Candidatus Nitrosopumilus piranensis strain D3C*	100.000	0.971
GCF_000956175.1	Reference	*Candidatus Nitrosopumilus adriaticus strain NF5*	100.000	0.000
GCF_002906215.1	Reference	*Candidatus Nitrosocaldus islandicus isolate 3F*	99.029	0.000
GCF_900065925.1	Reference	*Candidatus Nitrosotalea devanaterra*	98.544	0.000
GCF_900167955.1	Reference	*Candidatus Nitrosotalea bavarica isolate SBT1*	97.573	1.942
GCF_900248165.1	Reference	*Candidatus Nitrosocaldus cavascurensis strain SCU2*	99.029	0.000
GCF_900696045.1	Reference	*Candidatus Nitrosocosmicus franklandus C13*	98.058	1.942
GCA_000024305.1	Assembly	*Sulfolobus islandicus L.D.8.5*	99.405	0.000
GCA_000246735.1	Assembly	uncultured marine group II euryarchaeote	83.200	0.000
GCA_000698785.1	Assembly	*Nitrososphaera viennensis EN76*	100.000	0.971
GCA_000802205.2	Assembly	*Candidatus Nitrosocosmicus oleophilus strain MY3*	98.058	0.971

GCA_001917865.1	Assembly	*Candidatus Nitrososphaera sp. 13 1 40CM 48 12*	48.058	1.942
GCA_003056285.1	Assembly	*Aigarchaeota archaeon NZ13 MG1*	99.029	0.485
GCA_003702485.1	Assembly	*Candidatus Nitrosopelagicus sp. Isolate GoM MAG1*	75.902	3.883
GCA_900143675.1	Assembly	*Candidatus Nitrosotalea sinensis Nd2*	99.515	0.971
GCA_900177045.1	Assembly	*Nitrosotalea sp. CS isolate NCS1*	99.515	0.000
IMG_2263082000	[a] SAG	Thaumarchaeota archaeon strain DS1	89.159	0.971
IMG_2264867067	SAG	*Candidatus Iainarchaeum andersonii SCGC AAA011-E11*	90.187	0.000
IMG_2264867070	SAG	*Nanoarchaeota archaeon SCGC AAA011-G17*	73.113	0.000
IMG_2264867229	SAG	Aigarchaeota archaeon JGI 0000106-J15	56.553	0.485
IMG_2513237066	SAG	Marine Group I crenarchaeote sp. SCGC AAA288-I14	78.560	1.942
IMG_2513237068	SAG	Marine Group I crenarchaeote sp. SCGC AAA007-N19	70.276	0.000
IMG_2524023104	SAG	Marine Group I thaumarchaeote sp. SCGC AAA288-P02	49.035	8.495
IMG_2527291500	SAG	Marine Group I thaumarchaeote SCGC AAA007-O23	97.330	2.091
IMG_2527291509	SAG	Aigarchaeota archaeon SCGC AAA471-B22	82.649	4.369
IMG_2545555825	[b] MAG	Aigarchaeota archaeon strain OS1	76.805	0.000

IMG_2558309099	MAG	Thaumarchaeota bin Fn1	97.249	2.913
IMG_2619618950	SAG	Aigarchaeota archaeon JGI MDM2 JNZ-1-K18	75.935	0.935
IMG_2654587960	MAG	Miscellaneous Crenarchaeota group-6 archaeon AD8-1	95.794	4.206
IMG_2654588083	MAG	Miscellaneous Crenarchaeota group-15 archaeon DG-45	73.364	13.012
IMG_2708742552	MAG	Thaumarchaeota archaeon RBG 16 49 8	71.247	0.000
IMG_2718217642	MAG	Unclassified Aigarchaeota JZ bin 40	98.058	0.000
IMG_2721755844	MAG	Unclassified Thaumarchaeota DRTY-7 bin 36	93.689	0.971

[a] SAG: single-cell amplified genome

[b] MAG: metagenome-assembled genome

Table S8. Nitrogen model constants.

Parameter	Description	Value	Reference / Notes
k_{NO3}	present day ocean nitrate reservoir	$4.35\text{x}10^{16}$ molN	(Lenton and Daines 2018)
k_{NH4}	present day ocean ammonium reservoir	$1.4\text{x}10^{13}$ molN	from [NH_4] ~0.01 μM, ocean mass $1.4\text{x}10^{21}$ kg
k_{NFix}	present day nitrogen fixation flux	$8.6\text{x}10^{12}$ molN yr^{-1}	(Lenton and Daines 2018)
k_{def}	present day deficit of N below Redfield ratio (N:P=16) in global ocean	$6.086\text{x}10^{15}$ molN	from k_{NO3}, k_{NH4} and $3.1\text{x}10^{15}$ molP present day ocean reservoir
k_{mix}	global ocean upwelling rate	0.006 yr^{-1}	(Tyrrell 1999)
k_{uptake}	global efficiency of conversion of upwelled nutrient into export production	0.5	(Lenton and Daines 2018)
k_{anox}	controls sharpness of transition between oxic and anoxic deep ocean	12	(Lenton and Daines 2018)
E_{max}	present day maximum potential export production	$2.69684\text{x}10^{14}$ molN yr^{-1}	from k_{mix}, k_{NO3}, k_{NH4}, k_{NFix}
f_0	present day anoxic fraction	0.00299	(Lenton and Daines 2018)
k_{DeN}	half of present day denitrification flux	$4.3\text{x}10^{12}$ molN yr^{-1}	(Lenton and Daines 2018)
K_m^{AOA}	AOA nitrification half-saturation constant	$2.1\text{x}10^{14}$ molNH_4 ([NH_4]~0.15 μM)	(Martens-Habbena et al. 2009)
k_{Nit}^{AOA}	AOA nitrification rate constant (average)	0.429 yr^{-1}	to achieve steady state $1.4\text{x}10^{13}$ molNH_4 today
K_m^{LAN}	Low affinity nitrification half-saturation constant (based on AOB)	$1.19\text{x}10^{16}$ molNH_4 ([NH_4]~8.5 μM)	(Prosser and Nicol 2012)
k_{Nit}^{LAN}	Low affinity nitrification rate constant (average)	1 yr^{-1}	(Fennel et al. 2005)

Supplementary Datasets

Dataset S1. Seventy markers sequences

Dataset S2. Ninety concatenated marker protein sequences for phylogenomic tree

Dataset S3. Phylogenomic tree (newick format)

Dataset S4. AmoA gene sequences for phylogenetic tree

Dataset S5. AmoA gene tree (newick format)

SI References

Altschul SF, Madden TL, Schaffer AA, Zhang J, Zhang Z, Miller W, Lipman DJ. 1997. Gapped BLAST and PSI-BLAST: a new generation of protein database search programs. Nucleic Acids Res. 25:3389-3402.

Alves RJE, Minh BQ, Urich T, von Haeseler A, Schleper C. 2018. Unifying the global phylogeny and environmental distribution of ammonia-oxidising archaea based on amoA genes. Nat. Commun. 9:1517.

Bachan A, Kump LR. 2015. The rise of oxygen and siderite oxidation during the Lomagundi Event. Proceedings of the National Academy of Sciences 112:6562-6567.

Bekker A, Holland H. 2012. Oxygen overshoot and recovery during the early Paleoproterozoic. Earth and Planetary Science Letters 317:295-304.

Bekker A, Holland H, Wang P-L, Rumble D, Stein H, Hannah J, Coetzee L, Beukes N. 2004. Dating the rise of atmospheric oxygen. Nature 427:117-120.

Blank CE. 2011. An Expansion of Age Constraints for Microbial Clades that Lack a Conventional Fossil Record Using Phylogenomic Dating. J. Mol. Evol. 73:188-208.

Blank CE. 2009a. Not so old Archaea–the antiquity of biogeochemical processes in the archaeal domain of life. Geobiology 7:495-514.

Blank CE. 2009b. Phylogenomic dating—a method of constraining the age of microbial taxa that lack a conventional fossil record. Astrobiology 9:173-191.

Campbell IH, Allen CM. 2008. Formation of supercontinents linked to increases in atmospheric oxygen. Nature Geoscience 1:554-558.

Caporaso JG, Kuczynski J, Stombaugh J, Bittinger K, Bushman FD, Costello EK, Fierer N, Pena AG, Goodrich JK, Gordon JI, et al. 2010. QIIME allows analysis of high-throughput community sequencing data. Nat. Methods 7:335-336.

Criscuolo A, Gribaldo S. 2010. BMGE (Block Mapping and Gathering with Entropy): a new software for selection of phylogenetic informative regions from multiple sequence alignments. BMC Evol. Biol. 10:210.

Daines SJ, Mills BJ, Lenton TM. 2017. Atmospheric oxygen regulation at low Proterozoic levels by incomplete oxidative weathering of sedimentary organic carbon. Nat. Commun. 8:14379.

de Vienne DM, Giraud T, Martin OC. 2007. A congruence index for testing topological similarity

between trees. Bioinformatics 23:3119-3124.

Douzery EJ, Snell EA, Bapteste E, Delsuc F, Philippe H. 2004. The timing of eukaryotic evolution: does a relaxed molecular clock reconcile proteins and fossils? Proceedings of the National Academy of Sciences 101:15386-15391.

Eme L, Sharpe SC, Brown MW, Roger AJ. 2014. On the age of eukaryotes: evaluating evidence from fossils and molecular clocks. Cold Spring Harb. Perspect. Biol. 6:a016139.

Fennel K, Follows M, Falkowski PG. 2005. The co-evolution of the nitrogen, carbon and oxygen cycles in the Proterozoic ocean. Am. J. Sci. 305:526-545.

Gumsley AP, Chamberlain KR, Bleeker W, Soderlund U, de Kock MO, Larsson ER, Bekker A. 2017. Timing and tempo of the Great Oxidation Event. Proc. Natl. Acad. Sci. U. S. A. 114:1811-1816.

Hoffman PF, Abbot DS, Ashkenazy Y, Benn DI, Brocks JJ, Cohen PA, Cox GM, Creveling JR, Donnadieu Y, Erwin DH. 2017. Snowball Earth climate dynamics and Cryogenian geology-geobiology. Science Advances 3:e1600983.

Krause AJ, Mills BJ, Zhang S, Planavsky NJ, Lenton TM, Poulton SW. 2018. Stepwise oxygenation of the Paleozoic atmosphere. Nat. Commun. 9:4081.

Kumar S, Stecher G, Li M, Knyaz C, Tamura K. 2018. MEGA X: molecular evolutionary genetics analysis across computing platforms. Mol. Biol. Evol. 35:1547-1549.

Lanfear R, Frandsen PB, Wright AM, Senfeld T, Calcott B. 2017. PartitionFinder 2: New Methods for Selecting Partitioned Models of Evolution for Molecular and Morphological Phylogenetic Analyses. Mol. Biol. Evol. 34:772-773.

Lenton TM, Daines SJ. 2017. Biogeochemical transformations in the history of the ocean. Ann. Rev. Mar. Sci. 9:31-58.

Lenton TM, Daines SJ. 2018. The effects of marine eukaryote evolution on phosphorus, carbon and oxygen cycling across the Proterozoic–Phanerozoic transition. Emerging Topics in Life Sciences 2:267-278.

Lenton TM, Daines SJ, Mills BJ. 2018. COPSE reloaded: an improved model of biogeochemical cycling over Phanerozoic time. Earth-Sci. Rev. 178:1-28.

Martens-Habbena W, Berube PM, Urakawa H, de la Torre JR, Stahl DA. 2009. Ammonia oxidation kinetics determine niche separation of nitrifying Archaea and Bacteria. Nature 461:976-U234.

Mello B, Tao Q, Tamura K, Kumar S. 2016. Fast and accurate estimates of divergence times from big data. Mol. Biol. Evol. 34:45-50.

Monteiro F, Pancost R, Ridgwell A, Donnadieu Y. 2012. Nutrients as the dominant control on the spread of anoxia and euxinia across the Cenomanian-Turonian oceanic anoxic event (OAE2): Model-data comparison. Paleoceanography 27.

Nguyen L-T, Schmidt HA, Von Haeseler A, Minh BQ. 2015. IQ-TREE: a fast and effective stochastic algorithm for estimating maximum-likelihood phylogenies. Mol. Biol. Evol. 32:268-274.

Och LM, Shields-Zhou GA. 2012. The Neoproterozoic oxygenation event: environmental perturbations and biogeochemical cycling. Earth-Sci. Rev. 110:26-57.

Papadopoulos JS, Agarwala R. 2007. COBALT: constraint-based alignment tool for multiple protein sequences. Bioinformatics 23:1073-1079.

Parfrey LW, Lahr DJ, Knoll AH, Katz LA. 2011. Estimating the timing of early eukaryotic diversification with multigene molecular clocks. Proceedings of the National Academy of Sciences 108:13624-13629.

Parks DH, Imelfort M, Skennerton CT, Hugenholtz P, Tyson GW. 2015. CheckM: assessing the quality of microbial genomes recovered from isolates, single cells, and metagenomes. Genome Res. 25:1043-1055.

Pester M, Rattei T, Flechl S, Grongroft A, Richter A, Overmann J, Reinhold-Hurek B, Loy A, Wagner M. 2012. amoA-based consensus phylogeny of ammonia-oxidizing archaea and deep sequencing of amoA genes from soils of four different geographic regions. Environ. Microbiol. 14:525-539.

Petitjean C, Deschamps P, Lopez-Garcia P, Moreira D. 2015. Rooting the Domain Archaea by Phylogenomic Analysis Supports the Foundation of the New Kingdom Proteoarchaeota. Genome Biol. Evol. 7:191-204.

Petitjean C, Moreira D, Lopez-Garcia P, Brochier-Armanet C. 2012. Horizontal gene transfer of a chloroplast DnaJ-Fer protein to Thaumarchaeota and the evolutionary history of the DnaK chaperone system in Archaea. BMC Evol. Biol. 12.

Prosser JI, Nicol GW. 2012. Archaeal and bacterial ammonia-oxidisers in soil: the quest for niche specialisation and differentiation. Trends Microbiol. 20:523-531.

Robinson O, Dylus D, Dessimoz C. 2016. Phylo. io: interactive viewing and comparison of large phylogenetic trees on the web. Mol. Biol. Evol. 33:2163-2166.

Rooney AD, Strauss JV, Brandon AD, Macdonald FA. 2015. A Cryogenian chronology: Two long-lasting synchronous Neoproterozoic glaciations. Geology 43:459-462.

Stamatakis A. 2014. RAxML version 8: a tool for phylogenetic analysis and post-analysis of large phylogenies. Bioinformatics 30:1312-1313.

Tamura K, Battistuzzi FU, Billing-Ross P, Murillo O, Filipski A, Kumar S. 2012. Estimating divergence times in large molecular phylogenies. Proceedings of the National Academy of Sciences 109:19333-19338.

Tamura K, Tao Q, Kumar S. 2018. Theoretical foundation of the RelTime method for estimating divergence times from variable evolutionary rates. Mol. Biol. Evol. 35:1770-1782.

Tang H, Chen Y. 2013. Global glaciations and atmospheric change at ca. 2.3 Ga. Geoscience Frontiers 4:583-596.

Tao Q, Tamura K, Mello B, Kumar S. 2020. Reliable confidence intervals for RelTime estimates of evolutionary divergence times. Mol. Biol. Evol. 37:280-290.

Tria FDK, Landan G, Dagan T. 2017. Phylogenetic rooting using minimal ancestor deviation. Nature ecology & evolution 1:0193.

Tyrrell T. 1999. The relative influences of nitrogen and phosphorus on oceanic primary production. Nature 400:525.

Ueno Y, Johnson MS, Danielache SO, Eskebjerg C, Pandey A, Yoshida N. 2009. Geological sulfur isotopes indicate elevated OCS in the Archean atmosphere, solving faint young sun paradox. Proc. Natl. Acad. Sci. U. S. A. 106:14784-14789.

Valley JW, Cavosie AJ, Ushikubo T, Reinhard DA, Lawrence DF, Larson DJ, Clifton PH, Kelly TF, Wilde SA, Moser DE. 2014. Hadean age for a post-magma-ocean zircon confirmed by atom-probe tomography. Nature Geoscience 7:219-223.

Warke MR, Di Rocco T, Zerkle AL, Lepland A, Prave AR, Martin AP, Ueno Y, Condon DJ, Claire MW. 2020. The Great Oxidation Event preceded a Paleoproterozoic "snowball Earth". Proc. Natl. Acad. Sci. U. S. A. 117:13314-13320.

Wolfe JM, Fournier GP. 2018. Horizontal gene transfer constrains the timing of methanogen evolution. Nature ecology & evolution 2:897.

Yang Z. 2007. PAML 4: phylogenetic analysis by maximum likelihood. Mol. Biol. Evol. 24:1586-1591.
